# Actin Is Required for Cellular Development and Virulence of *Botrytis cinerea* via the Mediation of Secretory Proteins

**DOI:** 10.1128/mSystems.00732-19

**Published:** 2020-02-25

**Authors:** Hua Li, Zhanquan Zhang, Guozheng Qin, Chang He, Boqiang Li, Shiping Tian

**Affiliations:** a Key Laboratory of Plant Resources, Institute of Botany, The Innovative Academy of Seed Design, Chinese Academy of Sciences, Beijing, China; b University of Chinese Academy of Sciences, Beijing, China; c Key Laboratory of Post-Harvest Handling of Fruits, Ministry of Agriculture of China, Beijing, China; University of California San Diego

**Keywords:** actin, *Botrytis cinerea*, pathogenesis, secretome

## Abstract

Actin is a vital component of the cytoskeleton of living cells and is involved in several complex processes. However, its functions in plant-pathogenic fungi are largely unknown. In this paper, we found that deletion of the Botrytis cinerea actin gene *bcactA* reduced growth and sporulation of *B. cinerea* and lowered virulence. Based on iTRAQ (isobaric tags for relative and absolute quantification)-based proteomic analysis, we compared changes of the secretome in Δ*bcactA* and wild-type strains. A total of 40 proteins exhibited significant differences in abundance in Δ*bcactA* mutants compared with the wild type. These proteins included 11 down-accumulated cell wall-degrading enzymes (CWDEs). Among them, two CWDEs, cellobiohydrolase (BcCBH) and β-endoglucanase (BcEG), were found to contribute to the virulence of *B. cinerea*, indicating that *bcactA* plays a crucial role in regulating the secretion of extracellular virulence factors. These findings unveil previously unknown functions of BcactA to mediate the virulence of *B. cinerea* and provide new mechanistic insights into the role of BcactA in the complex pathogenesis of *B. cinerea*.

**IMPORTANCE** The cytoskeleton is an important network that exists in cells of all domains of life. In eukaryotic cells, actin is a vital component of the cytoskeleton. Here, we report that BcactA, an actin protein in *B. cinerea*, can affect the growth, sporulation, and virulence of *B. cinerea*. Furthermore, iTRAQ-based proteomic analysis showed that BcactA affects the abundance of 40 extracellular proteins, including 11 down-accumulated CWDEs. Among them, two CWDEs, cellobiohydrolase (BcCBH) and β-endoglucanase (BcEG), contributed to the virulence of *B. cinerea*, indicating that *bcactA* plays a crucial role in regulating extracellular virulence factors. These findings unveil previously unknown functions of BcactA in mediating growth, sporulation, and virulence of *B. cinerea*.

## Abstract (precis)

The cytoskeleton is an important network that exists in cells of all domains of life. In eukaryotic cells, actin is a vital component of the cytoskeleton. Here, we report that BcactA, an actin protein in *B. cinerea*, can affect the growth, sporulation, and virulence of *B. cinerea*. Furthermore, iTRAQ-based proteomic analysis showed that BcactA affects the abundance of 40 extracellular proteins, including 11 down-accumulated CWDEs. Among them, two CWDEs, cellobiohydrolase (BcCBH) and β-endoglucanase (BcEG), contributed to the virulence of *B. cinerea*, indicating that *bcactA* plays a crucial role in regulating extracellular virulence factors. These findings unveil previously unknown functions of BcactA in mediating growth, sporulation, and virulence of *B. cinerea*.

## INTRODUCTION

The cytoskeleton is an extremely highly organized but complex and dynamic network that exists in cells of all domains of life, including archaea, bacteria, and eukaryotes. In eukaryotic cells, it is mainly composed of microfilaments, microtubules, and intermediate filaments. Actin is a vital component of microfilaments, and many actin isoforms exist. In mammals, there are three types of actin, namely, α-actin, β-actin, and γ-actin. Deficient mutants of some actin isoforms are lethal, while others are viable. In mice, whole-body β-actin-knockout (Actb^−/−^) mutants are lethal, while whole-body γ-actin-null (Actg1^−/−^) mutants are not (1). In plants, several actin proteins have been discovered. Misexpression of *act1* in vegetative tissues can cause dwarfing of Arabidopsis thaliana (2), while the *act2-1* mutant exhibits no phenotypic distinction from the wild type (WT) when grown in soil (3). In filamentous fungi, three high-order F-actin structures exist with distinct functions: actin patches, cables, and rings. Actin patches are peripheral punctate structures present at subapical regions, where the endocytic machinery is located (4). The localization of actin patches is indicative of actin’s function in endocytosis and exocytosis at the hyphal tip and in coupling those functions to maintain tip growth (5–7). Actin cables predominantly localize at the apex of hyphae and form tracks for myosin V-dependent polarized secretion and organelle transport (8–10). Actin rings participate in septum formation and are essential for cytokinesis in budding yeast and pathogenesis in Magnaporthe oryzae (11–13). Actin or actin-related proteins are widely involved in the pathogenicity and secretion of fungi. The F-actin capping protein is important for the hyphal growth and virulence of Botrytis cinerea (14). Disruptions of the actin cytoskeleton in Aspergillus nidulans can lead to the inhibition of enzyme secretion via blockade of secretory vesicle transportation (15).

*B. cinerea* is a major phytopathogenic fungus that has been classified as the second most devastating plant pathogen (16). It invades more than 1,000 plant species, particularly fresh horticultural crops, during cultivation, storage, and distribution, leading to huge economic losses of $10 to $100 billion annually worldwide (16–18). In the past few years, significant efforts have been focused on effective control by exploring the molecular mechanisms of the pathogenicity of *B. cinerea*. The secretory proteins of *B. cinerea* comprise various virulence factors that facilitate successful host tissue penetration and colonization (19). Our previous studies have shown that *B. cinerea* is able to secrete a variety of enzymes and metabolites that can kill host cells during the infection process (20) and even adjust its secretome in response to changes in ambient pH values (21). The Rab family protein Bcsas1 also plays an important role in hyphal growth and virulence regulation in *B. cinerea* by affecting vesicle transport and protein secretion (22). Therefore, secretome analysis can provide new insights into the role of extracellular proteins in pathogenic invasion. Considering that actin is a cytoskeleton protein reportedly involved in the secretion of enzymes secretion of A. nidulans (15), it may also be involved in the secretion of virulence factors in *B. cinerea*. Among the actin proteins, the expression of BcactA was observed to be increased for the first 4 days when bean leaves were inoculated with *B. cinerea* and thereafter decreased (23).

Proteomics have played a dominant role in the study of specific proteins involved in the virulence of plant pathogens (24). Although next-generation and third-generation high-throughput sequencing have developed rapidly and have a strong advantage in determining global changes at the transcription level, they are insufficient in predicting changes at the protein level, because divergence exists between gene transcription and protein expression. Besides gene transcription, protein expression can also be affected by transcription regulation and protein translation (25). In addition, proteomics, such as isobaric tags for relative and absolute quantification (iTRAQ)-based quantitative proteomic analysis, is more efficient in investigating the subproteome, as proteins can be carefully isolated before analysis. We have successfully used proteomics in the past to investigate the subproteome of both nuclear proteins in tomato fruits at different ripening stages (26) and tonoplast proteins in apple fruits at different periods of senescence (27).

In this study, the mutant of the cytoskeleton protein BcactA was constructed. Deletion of *bcactA* resulted in reduced growth rate, sporulation, and virulence of *B. cinerea.* To further unveil the downstream proteins regulated by BcactA, iTRAQ-based quantitative proteomic analysis was conducted. Forty differentially expressed secretory proteins were identified as being associated with the *ΔbcactA* mutant. Among them, BcCBH and BcEG were shown to be involved in the regulation of *B. cinerea* virulence. These results reveal a previously unknown function of actin to control both the virulence of a phytopathogen and its associated involved mechanisms.

## RESULTS

### BcactA is required for vegetative growth and sporulation.

**FIG 1:**
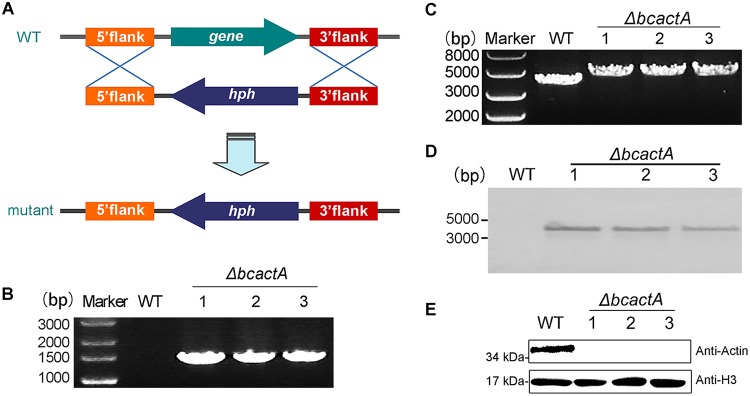
Generation of Δ*bcactA* mutants. (A) Replacement strategy for deletion of the *bcactA* gene. The 5′ flank and 3′ flank of *bcactA* were amplified and ligated into pLOB7 to flank the hygromycin resistance cassette to obtain the replacement vectors. Deletion mutants were generated by replacing the *bcactA* gene with the hygromycin resistance cassette through transformation of the protoplasts of the wild-type (WT) strain. (B) PCR diagnosis of Δ*bcactA* mutants. PCR was performed using the primer pair *bcactA*-homo-up/HPH-det, to ensure that homologous recombination occurred at the target site. (C) Diagnosis of the homozygotes of Δ*bcactA* mutants. PCR was performed with the primer pair *bcactA*-homo-up/*bcactA*-R-down, to ensure that the mutants were homozygous with no wild-type bands. (D) Southern blot analysis of WT and Δ*bcactA* strains. The genomic DNA was digested with SacI and BamHI, separated in an agarose gel, and hybridized with a probe (a fragment on the hygromycin resistance cassette labeled with digoxigenin). Numbers represent different strains. (E) Immunoblotting analysis of BcactA protein in WT and Δ*bcactA* strains. Total cytoplasmic proteins were extracted from 2-day-old mycelia cultured in PDB and immunoblotted with commercially available antibody to actin (Abmart; M20011). Histone 3 (H3) was used as a loading control.

**FIG 2:**
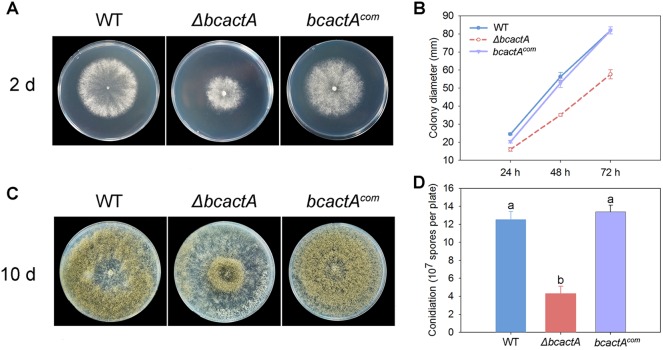
Phenotype detection of Δ*bcactA* mutants. (A) Colony morphology of wild-type (WT), Δ*bcactA*, and *bcactA^com^* strains after 48 h of growth on complete medium (CM) plates. (B) Statistical analysis of colony diameter. (C) Sporulation of WT, Δ*bcactA*, and *bcactA^com^* strains after 10 days of growth on CM plates. (D) Statistical analysis of sporulation. Strains were cultured at 22°C. Phenotype detection experiments were repeated three times with similar results, and the pictures shown are representative. Bars represent standard deviations (SD) of the means from three CM plates. Different letters in the same column indicate significant differences (*P* < 0.05).

The BcactA gene (Bcin16g02020) encodes a conserved actin protein. The amino acid sequence of BcactA shows high homology with the actin proteins in model organisms (see Fig. S1 in the supplemental material). To investigate the role of BcactA in the growth, colony morphology, and sporulation of *B. cinerea*, a knockout mutant of *bcactA* was generated by replacing the *bcactA* gene (Bcin16g02020) with a hygromycin resistance cassette through transformation of protoplasts of the wild-type strain (Fig. 1A). Three independent transformants were obtained through screening on selection medium supplemented with hygromycin B and subsequent PCR verification (Fig. 1B). After single spore isolation, the transformants were verified by PCR as homozygous (Fig. 1C) and further confirmed to be single-copy insertions by Southern blot analysis (Fig. 1D). To further confirm that the mutant indeed lost the actin protein, we conducted Western blot analysis with a commercial antibody to actin. The results indicated that in the Δ*bcactA* knockout mutant, the actin protein was completely lost (Fig. 1E and Fig. S2). To confirm the correlation between the phenotype of the Δ*bcactA* mutant and the inactivation of *bcactA*, complemented strains were constructed and verified by PCR (Fig. S3). The growth rate of the Δ*bcactA* mutant was reduced compared with that of the wild-type and complemented strains on complete medium (CM). The colony diameter of the mutant exhibited a 34% reduction at 24 h postinoculation (hpi), and a 36% reduction at 48 hpi, relative to the wild-type and complemented strains (Fig. 2B). The hyphae of the Δ*bcactA* mutant were more compact than those of the wild-type and complemented strains on CM (Fig. 2A). The sporulation of the Δ*bcactA* mutant was significantly reduced. After culture for 10 days on CM, the sporulation of the mutant was only 33% of that of the wild-type and complemented strains (Fig. 2D). The mutant spores were more likely to be distributed around the center of the plates, while the spores of the wild-type and complemented strains were more evenly distributed over the entire plates (Fig. 2C). These results show that BcactA plays a role in the growth, colony morphology, and sporulation of *B. cinerea*.

10.1128/mSystems.00732-19.1 FIG S1 Alignment of the protein sequence of BcactA with the actin protein sequences in model organisms. Download FIG S1, TIF file, 2.0 MB. Copyright © 2020 Li et al. 2020 Li et al. This content is distributed under the terms of the Creative Commons Attribution 4.0 International license.

10.1128/mSystems.00732-19.2 FIG S2 Western blot analysis of actin protein in different strains. (A) Immunoblot detection of actin protein in WT and actin mutants. A commercially available antibody to actin (Abmart; M20011) was used. (B) Histone 3 (H3) was used as a loading control. Histone 3 was detected using commercially available antibody to H3 (Agrisera; AS10710). (C) Coomassie brilliant blue staining was carried out as a loading control. Each sample was loaded with 40 μg protein. Download FIG S2, TIF file, 0.3 MB. Copyright © 2020 Li et al. 2020 Li et al. This content is distributed under the terms of the Creative Commons Attribution 4.0 International license.

10.1128/mSystems.00732-19.3 FIG S3 PCR diagnosis of the complemented lines of Δ*bcactA*, Δ*bccbh*, and Δ*bceg* mutants. Download FIG S3, TIF file, 0.1 MB. Copyright © 2020 Li et al. 2020 Li et al. This content is distributed under the terms of the Creative Commons Attribution 4.0 International license.

### BcactA participates in regulating the pathogenicity of *B. cinerea*.

**FIG 3:**
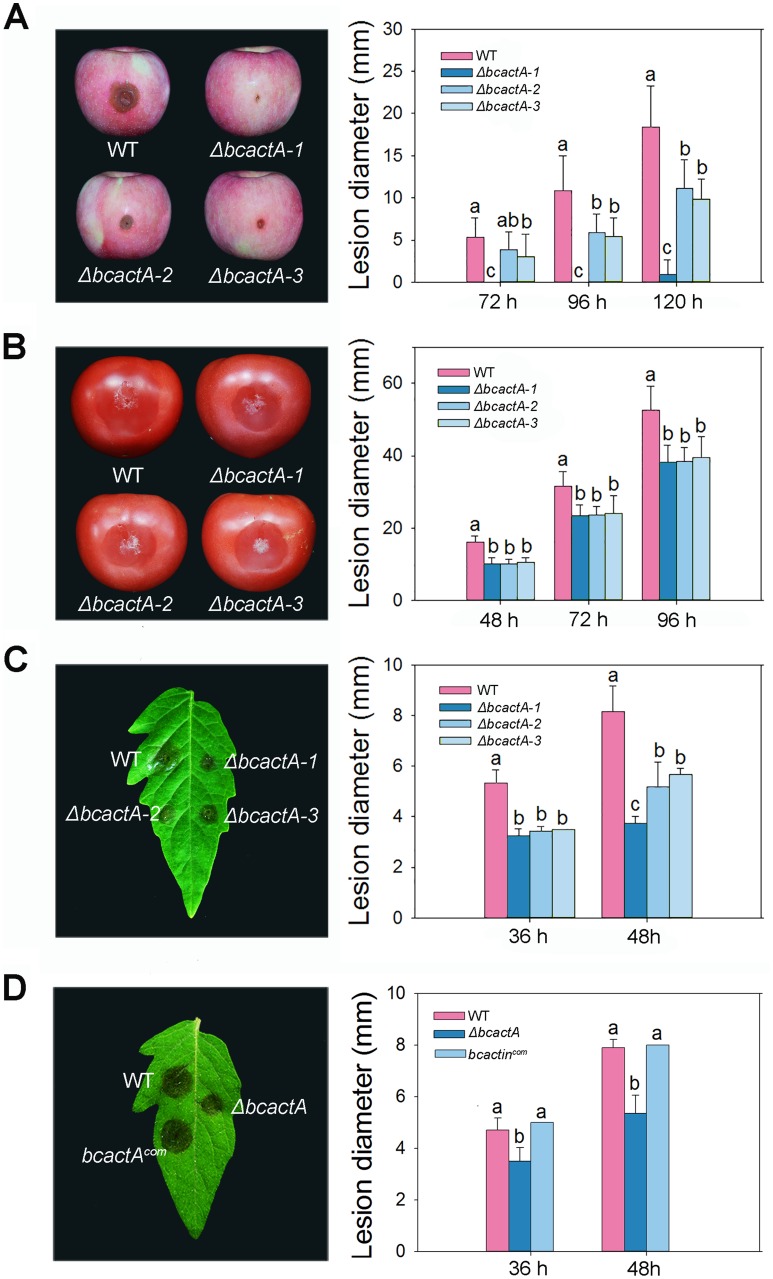
Virulence assays of wild-type (WT), Δ*bcactA*, and *bcactA^com^* strains in apple and tomato fruits, as well as detached tomato leaves. (A) Disease symptoms and statistical analysis of WT, Δ*bcactA1*, Δ*bcactA2*, and Δ*bcactA3* strains on apple fruits. Images were captured after 96 h of inoculation. (B) Disease symptoms and statistical analysis of WT, Δ*bcactA1*, Δ*bcactA2*, and Δ*bcactA3* strains on tomato fruits. Images were captured after 96 h of inoculation. (C) Disease symptoms and statistical analysis of WT, Δ*bcactA1*, Δ*bcactA2*, and Δ*bcactA3* strains on detached tomato leaves. Images were captured after 48 h of inoculation. (D) Disease symptoms and statistical results of lesion diameters in detached tomato leaves inoculated with WT, Δ*bcactA*, and *bcactA^com^* strains. Images were captured after 48 h of inoculation. Different letters in the same column indicate significant differences (*P* < 0.05).

To determine whether BcactA was involved in the regulation of pathogenicity in *B. cinerea*, apple and tomato fruits, as well as detached tomato leaves, were inoculated with the spores of Δ*bcactA* mutants. Δ*bcactA* mutants exhibited reduced virulence in different hosts (Fig. 3). At 96 hpi, apples inoculated with the Δ*bcactA1* mutant showed no lesions, while the WT-inoculated apples showed an average lesion size that reached 11 mm (Fig. 3A). Similarly, considerably reduced lesion sizes were observed in the Δ*bcactA1* mutant-inoculated tomato fruits in comparison with the WT-inoculated tomato fruits (Fig. 3B). At 48 hpi, lesion size was approximately 4 mm in the Δ*bcactA1* mutant-inoculated tomato leaves, compared with 8 mm in the WT-inoculated tomato leaves (Fig. 3C). The complemented strain of the Δ*bcactA1* mutant exhibited almost the same level of virulence as the WT (Fig. 3D). These results show that BcactA plays a role in the pathogenesis of *B. cinerea*.

### BcactA affects the contents of extracellular proteins.

**FIG 4:**
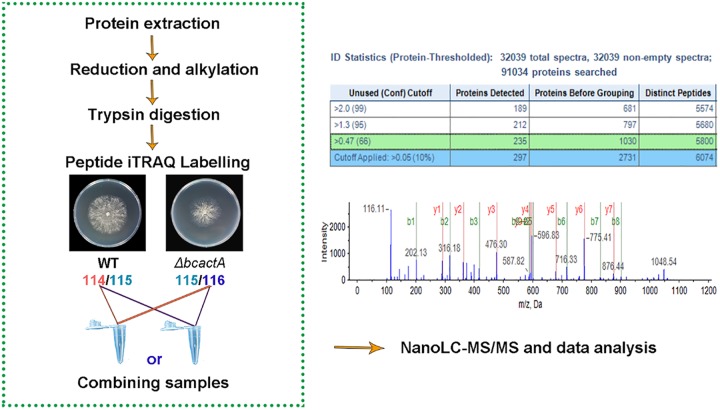
Workflow of quantitative proteomic analysis. Proteins were isolated from wild-type (WT) and Δ*bcactA* mutant strains and subjected to isobaric tags for relative and absolute quantification (iTRAQ) labeling coupled with nanoLC-MS/MS. Two biological repeats were conducted. Images of MS and data analysis were from one repeat.

**FIG 5:**
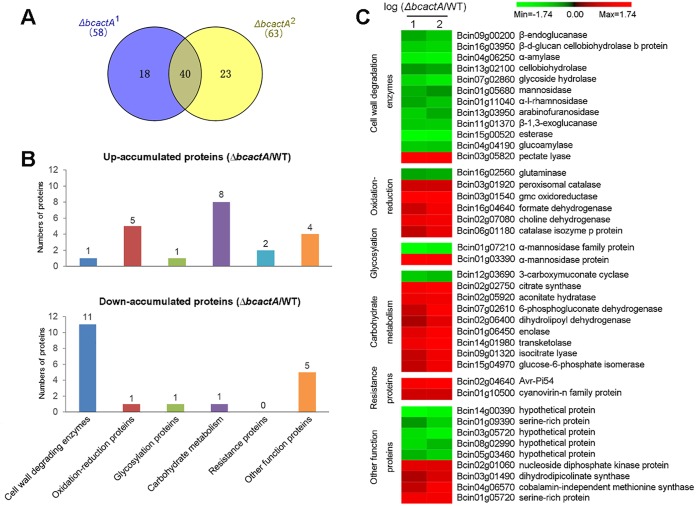
Analysis of differentially accumulated extracellular proteins in Δ*bcactA* mutants. (A) Venn diagram of differentially expressed proteins. Δ*bcactA*^1^ refers to the results of the first biological replication, while Δ*bcactA*^2^ refers to the results of the second biological replication. (B) Functional categories of up-accumulated and down-accumulated proteins in Δ*bcactA* mutant. (C) Expression patterns of proteins within each functional category. The logarithm of the protein ratio was used to measure the changes in protein abundance. Each row in the color heat map indicates a single protein. The gene identifiers (Bcin numbers) and functional annotations are shown. Red and green colors indicate up-accumulated and down-accumulated proteins, respectively, in Δ*bcactA* mutants versus WT.

To investigate the downstream targets of BcactA, extracellular proteins were extracted and iTRAQ-based quantitative proteomic analysis was used to analyze the secretome of the WT and Δ*bcactA* strains. Proteins were labeled with iTRAQ isobaric tags and subjected to nano-liquid chromatography–tandem mass spectrometry (nanoLC-MS/MS) analysis (Fig. 4). Totals of 220 and 232 proteins with a global false-discovery rate (FDR) below 1% were identified in the two biological replications, respectively, when searching against the *B. cinerea* protein database. A 2-fold cutoff was used as a determinant for proteins showing a significant change in abundance. The abundances of 58 and 63 proteins showed significant changes in the Δ*bcactA* mutant in the two biological replications, respectively, in comparison with the WT (40 proteins in common) (Fig. 5A). The up-accumulated and down-accumulated proteins in Δ*bcactA* mutants were classified into different functional categories, according to the blast2go tool, which included cell wall-degrading enzymes (CWDEs), oxidation-reduction proteins, glycosylation proteins, carbohydrate metabolism, resistance-associated proteins, and other functional proteins. The CWDEs were the most highly represented group among the categories (Fig. 5B). The ratios of iTRAQ reporter ion intensities, along with all relevant identification information for the differentially expressed proteins, are listed in Table 1.

**TABLE 1:** Differential proteins in *ΔbcactA* mutant versus the wild type

Function and accession no.^*a*^	Protein description	Log(Δ*bcactA*^1^:WT)^*b*^	Log(Δ*bcactA*^2^:WT)^*c*^	Unused protein score	Peptide (95%)^*d*^	% sequence coverage^*e*^
Cell wall degradation						
Bcin09g00200	β-Endoglucanase	−0.40	−0.57	21.40	24	37.53
Bcin16g03950	β-d-Glucan cellobiohydrolase b protein	−0.62	−0.68	13.63	9	32.44
Bcin04g06250	α-Amylase	−0.95	−1.04	48.98	47	53.20
Bcin13g02100	Cellobiohydrolase	−0.32	−0.38	84.18	81	68.41
Bcin07g02860	Glycoside hydrolase	−0.70	−0.90	8.02	5	15.06
Bcin01g05680	Mannosidase	−0.43	−0.30	17.91	9	16.98
Bcin01g11040	α-l-Rhamnosidase	−0.38	−0.54	33.32	24	32.35
Bcin13g03950	Arabinofuranosidase	−0.62	−0.40	92.26	95	73.12
Bcin11g01370	β-1,3-Exoglucanase	−0.58	−0.61	204.56	215	72.17
Bcin15g00520	Esterase	−1.26	−1.15	17.94	20	48.61
Bcin04g04190	Glucoamylase	−0.59	−0.57	83.15	68	59.84
Bcin03g05820	Pectate lyase	1.32	1.32	16.94	16	50.76
						
Oxidation-reduction						
Bcin16g02560	Glutaminase	−0.47	−0.51	57.49	52	57.68
Bcin03g01920	Peroxisomal catalase	0.78	0.78	33.78	26	43.03
Bcin03g01540	GMC oxidoreductase	1.56	1.65	354.50	418	84.87
Bcin16g04640	Formate dehydrogenase	0.74	1.28	41.14	27	51.72
Bcin02g07080	Choline dehydrogenase	1.14	1.29	131.40	127	57.82
Bcin06g01180	Catalase isozyme P protein	0.66	1.09	80.40	66	60.63
						
Glycosylation						
Bcin01g07210	α-Mannosidase family protein	−0.71	−0.56	15.71	10	17.53
Bcin01g03390	α-Mannosidase protein	0.68	0.64	23.99	16	16.96

Carbohydrate metabolism						
Bcin12g03690	3-Carboxymuconate cyclase	−0.59	−0.50	0.97	1	4.14
Bcin02g02750	Citrate synthase	1.03	1.29	17.09	11	30.08
Bcin02g05920	Aconitate hydratase	0.88	0.96	20.51	14	19.54
Bcin07g02610	6-Phosphogluconate dehydrogenase	0.54	1.17	16.69	15	25.48
Bcin02g06400	Dihydrolipoyl dehydrogenase	0.38	0.79	16.71	9	22.16
Bcin01g06450	Enolase	0.77	1.17	59.08	42	63.47
Bcin14g01980	Transketolase	0.93	1.30	39.63	22	32.41
Bcin09g01320	Isocitrate lyase	0.54	0.96	18.01	11	24.09
Bcin15g04970	Glucose-6-phosphate isomerase	0.53	0.96	29.94	28	35.70
						
Resistance associated						
Bcin02g04640	Avr-Pi54 protein	1.45	1.72	153.40	171	84.13
Bcin01g10500	Cyanovirin-N family protein	0.82	0.82	15.09	14	81.13
						
Other functions						
Bcin14g00390	Hypothetical protein	−1.51	−1.17	29.71	25	34.75
Bcin01g09390	Serine-rich protein	−0.36	−0.72	19.73	15	34.05
Bcin03g05720	Hypothetical protein	−0.86	−1.14	16.00	17	58.71
Bcin08g02990	Hypothetical protein	−0.83	−0.54	38.29	34	46.26
Bcin05g03460	Hypothetical protein	−0.51	−0.72	7.72	7	21.16
Bcin02g01060	Nucleoside diphosphate kinase protein	0.92	0.88	10.10	6	31.22
Bcin03g01490	Dihydrodipicolinate synthase protein	0.47	0.82	2.38	2	7.08
Bcin04g06570	Cobalamin-independent methionine synthase	0.61	1.26	21.30	15	20.60
Bcin01g05720	Serine-rich protein	1.16	1.16	102.30	100	57.48

a Accession number from Blast search on NCBI.

b The logarithm of the fold change of protein expression levels in *ΔbcactA* mutant versus the wild type from the first independent biological replicate. The meaningful cutoff was fixed at 2-fold corresponding to the logarithm of the iTRAQ ratio of >0.3 for upregulation and <−0.3 for downregulation, as mentioned in Materials and Methods.

c The logarithm of the fold change of protein expression levels in *ΔbcactA* mutant versus the wild type from the second independent biological replicate. The meaningful cutoff was fixed at 2-fold corresponding to the logarithm of the iTRAQ ratio of >0.3 for upregulation and <−0.3 for downregulation, as mentioned in Materials and Methods.

d Number of unique peptides identified (95% confidence).

e Amino acid sequence coverage for the identified proteins.

### CWDEs were decreased in Δ*bcactA* mutants.

To further analyze the 40 differentially expressed proteins identified in the Δ*bcactA* mutants, a heat map within each functional category was applied according to the expression patterns of identified proteins (Fig. 5C). Noticeably, 11 CWDEs were down-accumulated, except pectate lyase (BcPL; Bcin15g00520). The 11 CWDEs included mannosidase (BcMAN; Bcin01g05680), α-amylase (BcAMY; Bcin04g06250), glycoside hydrolase (BcGH; Bcin7g02860), β-1,3-exoglucanase (BcEXG; Bcin11g01370), glucoamylase (BcGA; Bcin04g04190), arabinofuranosidase (BcABF; Bcin13g03950), cellobiohydrolase (BcCBH; Bcin13g02100), endoglucanase (BcEG; Bcin09g00200), β-d-glucan cellobiohydrolase b (BcCBH-b; Bcin16g03950), α-l-rhamnosidase (BcRHA; Bcin01g11040), and esterase (BcEST; Bcin15g00520) (Fig. 5C). These results indicate that BcactA affected the level of CWDEs.

### Functional analysis of the identified CWDEs.

**FIG 6:**
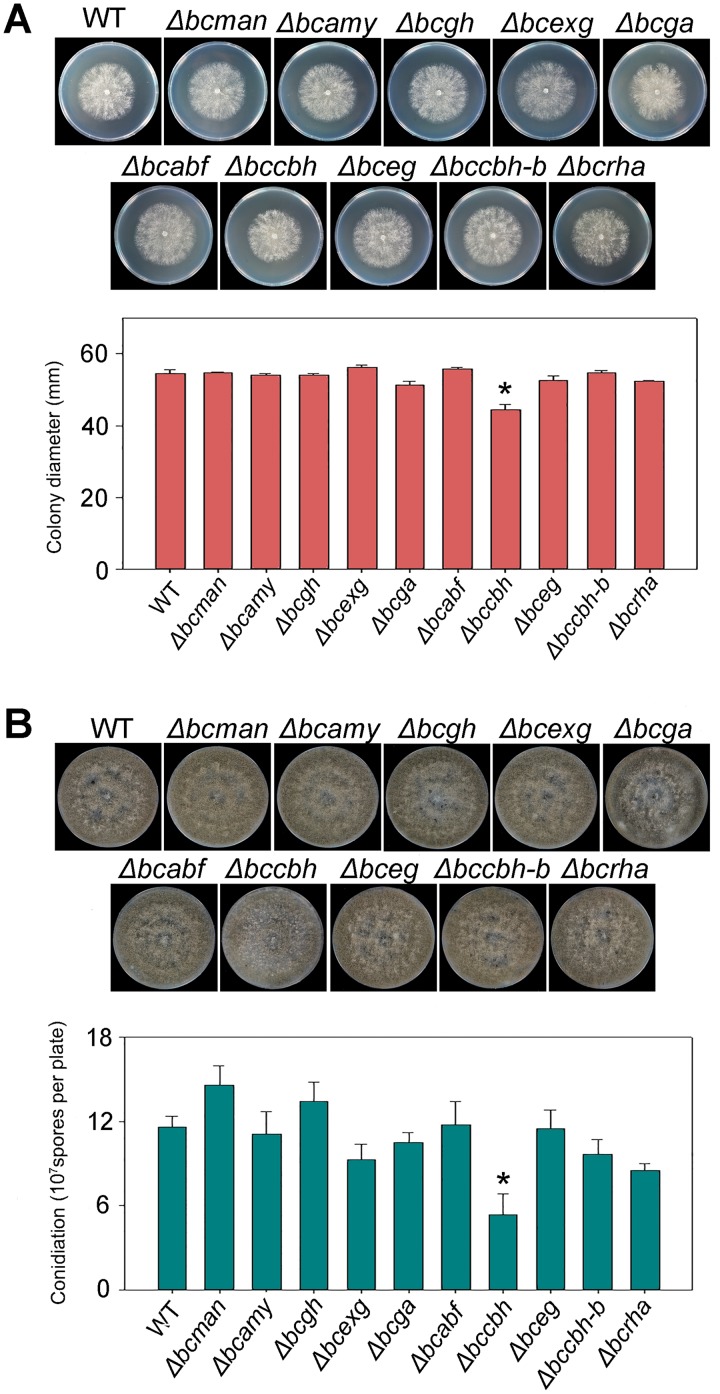
Phenotypic analysis of deletion mutants of 10 cell wall-degrading enzymes (CWDEs). (A) Colony morphology and colony diameters of wild-type (WT) and 10 CWDE mutant strains after 48 h of growth on complete medium (CM) plates. (B) Sporulating cultures and statistical analysis of sporulation of WT and 10 CWDE mutant strains after 10 days of growth on CM plates. Strains were cultured at 22°C. Data presented are the mean ± SD (*n* = 3). An asterisk indicates a significant difference from the WT (*P* < 0.05). *bcman*, mannosidase; *bcamy*, α-amylase; *bcgh*, glycoside hydrolase; *bcexg*, β-1,3-exoglucanase; *bcga*, glucoamylase; *bcabf*, arabinofuranosidase; *bccbh*, cellobiohydrolase; *bceg*, endoglucanase; *bccbh-b*, β-d-glucan cellobiohydrolase b; *bcrha*, α-l-rhamnosidase.

Among the 11 down-accumulated CWDEs, BcEST reportedly has no effect on the penetration or pathogenicity of *B. cinerea* (28). To further investigate the functions of the other 10 CWDEs (BcMAN, BcAMY, BcGH, BcEXG, BcGA, BcABF, BcCBH, BcEG, BcCBH-b, and BcRHA), knockout mutants of each gene were generated and verified to be homozygous mutants (Fig. S4 and S5). The growth rates of Δ*bcman*, Δ*bcamy*, Δ*bcgh*, Δ*bcexg*, Δ*bcga*, Δ*bcabf*, Δ*bceg*, Δ*bccbh*-*b*, and Δ*bcrha* mutants were almost the same as that of the WT, and only the growth of the Δ*bccbh* mutant was slower than that of the WT (Fig. 6A). Sporulation of the Δ*bccbh* mutant exhibited a significant reduction; however, that of other mutants showed no significant difference from the WT (Fig. 6B). Similarly, no significant differences were noted in colony morphology of all mutants in comparison with the WT, indicating that BcCBH affected hyphal growth of *B. cinerea.*

10.1128/mSystems.00732-19.4 FIG S4 PCR diagnosis of 10 cell wall-degrading enzyme (CWDE) mutants. PCR was performed with primer pairs binding outside the replacement fragment and within the resistance cassette, respectively. Detailed primer sequences are shown in Table S1. Numbers represent different strains. Download FIG S4, TIF file, 0.3 MB. Copyright © 2020 Li et al. 2020 Li et al. This content is distributed under the terms of the Creative Commons Attribution 4.0 International license.

10.1128/mSystems.00732-19.5 FIG S5 Diagnosis of the homozygotes of 10 cell wall-degrading enzyme (CWDE) mutants. PCR was performed with primer pairs with primer pairs binding outside the replacement fragment. Detailed primer sequences are shown in Table S1. Numbers represent different strains. Download FIG S5, TIF file, 0.3 MB. Copyright © 2020 Li et al. 2020 Li et al. This content is distributed under the terms of the Creative Commons Attribution 4.0 International license.

### BcCBH and BcEG are involved in the regulation of virulence in *B. cinerea*.

**FIG 7:**
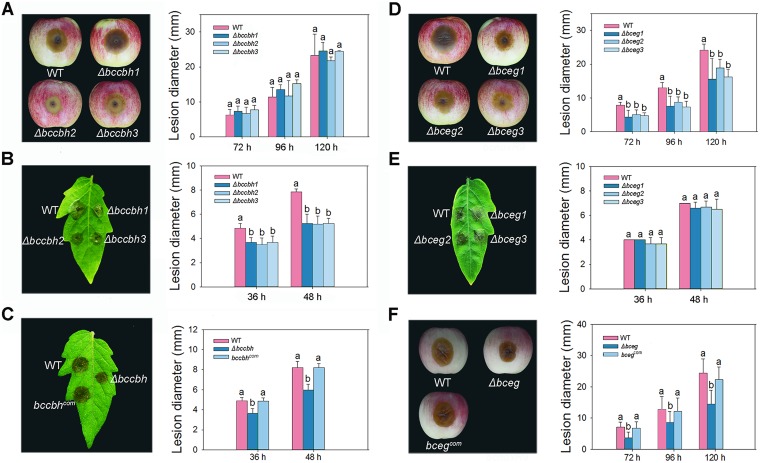
Virulence assays of Δ*bccbh* and Δ*bceg* strains in apple fruits and detached tomato leaves. (A) Disease symptoms (120 hpi) and statistical analysis of WT, Δ*bccbh1*, Δ*bccbh2*, and Δ*bccbh3* strains in apples. (B) Disease symptoms (48 hpi) and statistical analysis of WT, Δ*bccbh1*, Δ*bccbh2*, and Δ*bccbh3* strains in detached tomato leaves. (C) Disease symptoms (48 hpi) and statistical analysis of lesion diameters in detached tomato leaves inoculated with WT, *Δbccbh*, and *bccbh^com^* strains. (D) Disease symptoms (120 hpi) and statistical analysis of WT, Δ*bceg1*, Δ*bceg2*, and Δ*bceg3* strains in apples. (E) Disease symptoms (48 hpi) and statistical analysis of WT, Δ*bceg1*, Δ*bceg2*, and Δ*bceg3* strains in detached tomato leaves. (F) Disease symptoms (120 hpi) and statistical analysis of lesion diameters in apple fruits inoculated with WT, *Δbceg*, and *bceg^com^* strains. Different letters in the same column indicate significant differences (*P < *0.05).

To evaluate whether the 10 CWDEs were involved in the regulation of virulence of *B. cinerea*, spores of the mutants were collected and inoculated into apple fruits and detached tomato leaves. Compared with the WT-inoculated tomato leaves, lesion diameters were significantly smaller in the Δ*bccbh* mutant-inoculated tomato leaves (Fig. 7B). However, no significant differences were noted in lesion diameters on the Δ*bccbh* mutant-inoculated apple fruits (Fig. 7A). Nevertheless, the Δ*bceg* mutant exhibited a significant reduction in lesion diameters in the apple fruits (Fig. 7D) but not in detached tomato leaves (Fig. 7E). To confirm that virulence reduction of the mutants was due to the inactivation of corresponding genes, complemented strains were constructed and verified by PCR (Fig. S3). The complemented strains of the Δ*bccbh* and Δ*bceg* mutants were able to infect tomato leaves and apple fruits at the same rate as the WT (Fig. 7C and F). No significant differences in virulence were observed in other mutants in comparison with the WT (Fig. S6 and S7). These results suggested that two CWDEs, BcCBH and BcEG, were important for the pathogenesis of *B. cinerea.*

10.1128/mSystems.00732-19.6 FIG S6 Deletion of the other 8 CWDE genes did not affect virulence of *B. cinerea* in apple fruit. (A) Disease symptoms and statistical analysis of WT, Δ*bcman1*, Δ*bcman2*, and Δ*bcman3* strains in apple fruit. (B) Disease symptoms and statistical analysis of WT, Δ*bcamy1*, Δ*bcamy2*, and Δ*bcamy3* strains in apple fruit. (C) Disease symptoms and statistical analysis of WT, Δ*bcgh1*, Δ*bcgh2*, and Δ*bcgh3* strains in apple fruit. (D) Disease symptoms and statistical analysis of WT, Δ*bcexg1*, Δ*bcexg2*, and Δ*bcexg3* strains in apple fruit. (E) Disease symptoms and statistical analysis of WT, Δ*bcga1*, Δ*bcga2*, and Δ*bcga3* strains in apple fruit. (F) Disease symptoms and statistical analysis of WT, Δ*bcabf1*, Δ*bcabf2*, and Δ*bcabf3* strains in apple fruit. (G) Disease symptoms and statistical analysis of WT, Δ*bccbh-b1*, Δ*bccbh-b2*, and Δ*bccbh-b3* strains in apple fruit. (H) Disease symptoms and statistical analysis of WT, Δ*bcrha1*, Δ*bcrha2*, and Δ*bcrha3* strains in apple fruit. Photos were taken 120 h after inoculation. Columns with different letters at the same time are significantly different from each other, using the least significant difference test (*P* < 0.05). Download FIG S6, TIF file, 1.0 MB. Copyright © 2020 Li et al. 2020 Li et al. This content is distributed under the terms of the Creative Commons Attribution 4.0 International license.

10.1128/mSystems.00732-19.7 FIG S7 Deletion of the other 8 CWDE genes did not affect virulence of *B. cinerea* in detached tomato leaves. (A) Disease symptoms and statistical analysis of WT, Δ*bcman1*, Δ*bcman2*, and Δ*bcman3* strains in detached tomato leaves. (B) Disease symptoms and statistical analysis of WT, Δ*bcamy1*, Δ*bcamy2*, and Δ*bcamy3* strains in detached tomato leaves. (C) Disease symptoms and statistical analysis of WT, Δ*bcgh1*, Δ*bcgh2*, and Δ*bcgh3* strains in detached tomato leaves. (D) Disease symptoms and statistical analysis of WT, Δ*bcexg1*, Δ*bcexg2*, and Δ*bcexg3* strains in detached tomato leaves. (E) Disease symptoms and statistical analysis of WT, Δ*bcga1*, Δ*bcga2*, and Δ*bcga3* strains in detached tomato leaves. (F) Disease symptoms and statistical analysis of WT, Δ*bcabf1*, Δ*bcabf2*, and Δ*bcabf3* strains in detached tomato leaves. (G) Disease symptoms and statistical analysis of WT, Δ*bccbh-b1*, Δ*bccbh-b2*, and Δ*bccbh-b3* strains in detached tomato leaves. (H) Disease symptoms and statistical analysis of WT, Δ*bcrha1*, Δ*bcrha2*, and Δ*bcrha3* strains in detached tomato leaves. Photos were taken 48 h after inoculation. Columns with different letters at the same time are significantly different from each other, using the least significant difference test (*P* < 0.05). Download FIG S7, TIF file, 0.6 MB. Copyright © 2020 Li et al. 2020 Li et al. This content is distributed under the terms of the Creative Commons Attribution 4.0 International license.

## DISCUSSION

The cytoskeleton of living cells is a highly organized and dynamic network, which plays important roles in various cellular functions, including the regulation of cell polarity, endocytosis, protein secretion, septation, and organelle transport. In fungi, there are three forms of cytoskeleton, filamentous actin, microtubules, and septins, among which actin is a vital component. Actin exists in two forms, G-actin monomers and functional filamentous actin (F-actin). The transition to either of the two forms is closely related to its function (29). An NADPH-oxidase ortholog in yeast, Yno1p, was demonstrated to regulate actin cable formation in yeast cells (30). The regulatory subunit of NADPH-oxidase (NoxR) is required for the formation of a toroidal F-actin structure, which is essential for the protrusion of a rigid penetration peg in *M. oryzae* (11). In our previous study, we found that the protein abundance of actin was affected by NoxR in *B. cinerea* (31). Moreover, gene expression levels of eight NADPH oxidase (NOX) subunits, including *bcnoxR*, were higher in the Δ*bcactA* mutant (see Fig. S8 in the supplemental material), indicating that a complex and close association exists between BcnoxR and BcactA. The Δ*bcactA* mutant exhibited a reduction in growth rate, sporulation, and virulence in apple and tomato fruits and in detached tomato leaves (Fig. 2 and 3). The visibility of the Δ*bcactA* mutant was perhaps due to the functional redundancy of other actin-related proteins or other cytoskeletal forms.

10.1128/mSystems.00732-19.8 FIG S8 RT-qPCR analysis of the NOX subunit expression in WT and Δ*bcactA* strains. RT-qPCRs were performed on total RNA extracted from wild-type and Δ*bcactA* mutant strains 48 h after spores were cultured in liquid PDB. Relative gene expression values are presented after normalization against the tubulin gene, followed by normalization against WT. Gene expression values represent the mean ± SD from three biological replicates. Download FIG S8, TIF file, 0.2 MB. Copyright © 2020 Li et al. 2020 Li et al. This content is distributed under the terms of the Creative Commons Attribution 4.0 International license.

Actin plays a role in cell motility, maintenance of cell shape, and secretion (32, 33). F-actin cables extend from the plasma membrane into the cell and provide tracks for the targeted delivery of secretory vesicles (32, 33). In pathogenic fungi, the secretory proteins comprise virulence factors that facilitate successful host tissue penetration and colonization (19). Secretory proteins are important for the virulence of *B. cinerea*, as its appressorium is not rigid enough to rupture the host cell wall (34). *B. cinerea* utilizes different secretory proteins to invade host cells, depending on environmental conditions, such as differences in pH values. At pH 4, the secretome of *B. cinerea* favors proteolysis, which is essential for the degradation of the antifungal proteins secreted by the plant host (21, 35). At pH 6, the secretome of *B. cinerea* includes more CWDEs, which are helpful in decomposing the host cell wall to achieve full virulence (21, 36). Therefore, secretome analysis can provide new insights into extracellular protein function in pathogenic invasion. The iTRAQ-based quantitative proteomic analysis revealed characteristics such as high resolution, high throughput, accurate protein quantification, and repeatability (37). The technique has been used to characterize the dynamic changes of tonoplast proteins during apple fruit senescence (27). In this study, the secretome of the WT and Δ*bcactA* strains was examined through iTRAQ-based quantitative proteomic analysis, and 40 differentially expressed proteins were identified in the Δ*bcactA* mutant (Fig. 5). These proteins included oxidation-reduction-related proteins, resistance-associated proteins, and cell wall-degrading proteins, among others. Oxidation-reduction proteins are considered to be associated with pathogen invasion, because an oxidative burst usually occurs during infection (34, 38). We identified several oxidation-reduction proteins, including a Cu-Zn superoxide dismutase (Bcin03g03390) that was for the first time determined by proteomics. A deletion mutant of this gene results in reduced virulence in the host (39).

The CWDEs are important in the pathogenesis of phytopathogenic fungi (24). The plant cell wall, which is mainly composed of cellulose, xylan, pectin, and other polysaccharides, is an important barrier for effective defense against phytopathogenic fungi. The composition of the cell wall differs significantly among plant lineages and plant tissues (36). To penetrate the plant cell wall, phytopathogenic fungi produce a diverse array of CWDEs that are capable of degrading the main structural polysaccharide components of the cell wall (40). A total of 1,155 predicted genes encode the enzymes responsible for the creation, modification, or degradation of glycosidic bonds in *B. cinerea*, and 275 of those are predicted to be secretory proteins, as they contain extracellular signal peptide sequences (18). These secretory proteins have been frequently detected in comparative proteomics and are supposed to be essential virulence proteins (21, 41, 42). A variety of CWDEs have been proven to be essential for the virulence of *B. cinerea*. Endopolygalacturonase 1 (BcPG1) is involved in lesion expansion on apple fruits, tomato fruits, and leaves, while endopolygalacturonase 2 (BcPG2) is involved in both primary infection and lesion expansion of *B. cinerea* on tomato fruits and broad beans (43, 44). In addition, endo-β-1,4-xylanase (BcXYN11A), which can degrade xylan in the plant cell wall, is involved in the virulence of *B. cinerea* (45). In this study, we identified 11 CWDEs that were down-accumulated in the extracellular proteins of the *ΔbcactA* mutant (Fig. 5C). Cutinase A (BccutA) was previously reported as not essential for penetration of gerbera and tomato (28). The other 10 CWDEs are glycoside hydrolases (GHs), which are mainly associated in cellulose and hemicellulose degradation. We investigated the functions of the 10 GHs encoded by genes associated with growth, morphogenesis, and virulence of *B. cinerea*. The results indicated that two proteins, BcCBH and BcEG, were involved in the virulence of *B. cinerea.* The *Δbccbh* mutant showed a reduction in growth rate, sporulation, and virulence on detached tomato leaves, while the *Δbceg* mutant exhibited reduced virulence in apple fruits (Fig. 6 and 7). Other mutants showed almost the same virulence as the wild type, perhaps due to the redundancy of function provided by other enzymes, considering the large number of CWDEs in *B. cinerea*.

**FIG 8:**
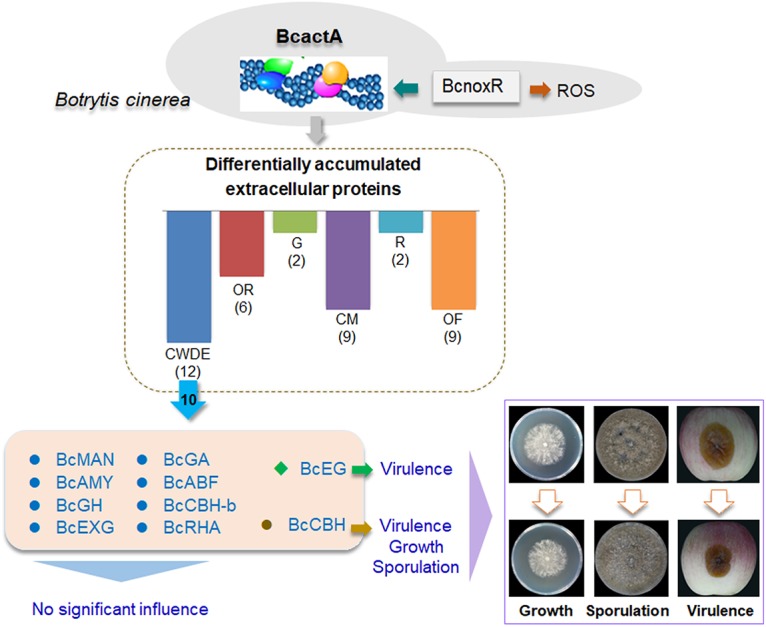
The mode of action of actin (BcactA) regulating the growth, development, and virulence of *B. cinerea*. BcnoxR regulates the actin cytoskeleton, and BcactA affects the secretion of extracellular proteins, which are divided into six groups according to their functions. Among 12 cell wall-degrading proteins, BcCBH is involved in growth, sporulation, and virulence, while BcEG is related to virulence in *B. cinerea*. CWDE, cell wall-degrading enzymes; OR, oxidation-reduction proteins; G, glycosylation proteins; CM, carbohydrate metabolism; R, resistance proteins; OF, other function proteins; BcMAN, mannosidase; BcAMY, α-amylase; BcGH, glycoside hydrolase; BcEXG, β-1,3-exoglucanase; BcGA, glucoamylase; BcABF, arabinofuranosidase; BcCBH, cellobiohydrolase; BcEG, endoglucanase; BcCBH-b, β-d-glucan cellobiohydrolase b; BcRHA, α-l-rhamnosidase; ROS, reactive oxygen species.

In conclusion, we found that the actin protein BcactA mediates growth, development, and virulence of *B. cinerea*, as well as the associated mechanisms. The secretion of a large number of extracellular CWDEs, including some critical virulence factors, is regulated by BcactA, which eventually affects the pathogenicity of *B. cinerea* (Fig. 8). These findings are beneficial for our understanding of the role of BcactA in the complex pathogenesis of *B. cinerea* and provide a novel insight into the regulation network of the actin cytoskeleton.

## MATERIALS AND METHODS

### Fungal strains and culture conditions.

*B. cinerea* (B05.10) was used as the recipient strain for the transformation experiments and as the wild-type control. The wild type and deletion mutants of *B. cinerea* were normally maintained at 22°C on CM, which contained 10 g glucose, 2 g peptone, 1 g yeast extract, 1 g Casamino Acids, 6 g NaNO_3_, 0.5 g KCl, 1.11 g MgSO_4_·7H_2_O, 1.5 g KH_2_PO_4_, and 0.05 g yeast nitrogen base in 1 liter of distilled water. For collection of spores, the strains of *B. cinerea* were first cultured on CM plates for 1 week at 22°C. Spores were then collected and diluted with sterile distilled water to 1 × 10^7^ spores ml^−1^, by counting with a hemocytometer under a microscope. For extracellular protein isolation, the spore suspension (1 ml) was added to potato dextrose broth (PDB; which contained 200 g peeled potatoes and 20 g glucose in 1 liter of distilled water) medium (100 ml) and cultured at 22°C for 24 h with shaking at 180 rpm. The mycelium was then harvested using four layers of gauze, washed thoroughly with sterile distilled water, and transferred to modified Czapek medium which contained 1% pectin from apples (Sigma, St. Louis, MO, USA), 0.2% NaNO_3_, 0.1% K_2_HPO_4_, 0.05% KCl, 0.05% MgSO_4_·7H_2_O, and 0.036 mM FeSO_4_·7H_2_O. After incubation at 22°C for 72 h with shaking at 180 rpm, the media were filtered with four layers of gauze and used for protein isolation.

### Generation of deletion mutants and complemented strains.

Approximately 1-kb flanking regions, proximal to the initiation codon and downstream of the termination codons of *bcactA*, *bcman*, *bcamy*, *bcgh*, *bcexg*, *bcga*, *bcabf*, *bccbh*, *bceg*, *bccbh-b*, and *bcrha*, were individually amplified. The amplified PCR products were cloned into pLOB7 (46), verified by sequencing, and introduced into protoplasts of the wild-type B05.10. Protoplast formation and transformation were performed according to the methods of a previous study (22). Briefly, the mycelium was suspended in 20 ml of 0.5% Glucanex solution (Sigma, St. Louis, MO) and incubated at 25°C and 100 rpm for 2 h to generate protoplasts. The transformation fragments were added into a suspension of 200 ml containing 2 × 10^7^ protoplasts. Thereafter 200 μl and 400 μl of 25% polyethylene glycol (PEG) 3350 (Sigma) in 50 mM CaCl_2_ and 10 mM Tris-HCl (pH 7.5) were added separately. The mixture was then transferred to SH agar, which contained 0.6 M sucrose, 5 mM HEPES, 1 mM (NH4)_2_HPO_4_, and 1% agar, to regenerate *B. cinerea* protoplasts. Transformants were initially selected on plates with hygromycin B and then checked using PCR.

Single-spore isolation was conducted to purify the deletion mutants. To verify single-copy genomic integration, Southern blot analysis was performed. Genomic DNA of wild type and three deletion mutants was digested with appropriate restriction enzyme pairs. Southern hybridization was performed with the right flank of the gene as a probe.

For complementation, the DNA fragments of specific genes, along with nucleotides approximately 2 kb upstream and 0.5 to 1 kb downstream, were amplified using wild-type genomic DNA and cloned into the pNAN-OGG vector with resistance to nourseothricin (23). The resulting plasmid was amplified, and the PCR products were used to transform the corresponding mutants. The transformants were initially selected on plates with nourseothricin and then identified by PCR. All primer pairs are listed in Table S1 in the supplemental material.

10.1128/mSystems.00732-19.9 TABLE S1 Primers used in vector construction. Download Table S1, DOCX file, 0.03 MB. Copyright © 2020 Li et al. 2020 Li et al. This content is distributed under the terms of the Creative Commons Attribution 4.0 International license.

### Isolation of extracellular proteins.

Extracellular proteins were isolated according to methods described previously (21). The separated media of wild-type and *ΔbcactA* strains were centrifuged three times at 20,000 × *g* for 30 min at 4°C to remove residual mycelia or other debris. The supernatant was mixed with an equal volume of Tris-HCl (pH 7.5)-buffered phenol by shaking for 30 min at 4°C and centrifuged at 10,000 × *g* for 40 min to eliminate contaminants. The lower phenol phase was mixed with 5 volumes of 0.1 M ammonium acetate in methanol and incubated at −20°C overnight to precipitate the proteins. Precipitated proteins were separated by centrifugation at 15,000 × *g* for 30 min at 4°C. The pellet was washed twice with prechilled 0.1 M ammonium acetate in methanol and twice with prechilled acetone. Protein pellets were air dried and stored at −80°C until further use.

### iTRAQ labeling and protein identification.

For secretome analysis, the extracellular proteins of WT and *ΔbcactA* strains were solubilized in the protein buffer, consisting of 100 mM triethylammonium bicarbonate (TEAB) and 0.04% (wt/vol) sodium dodecyl sulfate (SDS), pH 8.5. Protein concentrations were determined using the Bradford assay (47). Sixty micrograms of proteins from each sample was reduced by adding tris-(2-carboxyethyl)phosphine (TCEP) to a final concentration of 10 mM and incubated at 60°C for 1 h. Methyl methanethiosulfonate (MMTS) was added to each sample to a final concentration of 50 mM and incubated at 25°C for 10 min. The samples were then digested with 1.2 μg trypsin (Promega) at 37°C overnight. The tryptic peptides were labeled with an iTRAQ reagent 4-plex kit (Applied Biosystems) according to the manufacturer’s instructions. The iTRAQ tags were able to mark the free amino end. Two independent biological replications were performed. In the first biological replication, the wild-type and Δ*bcactA* strains were labeled with iTRAQ tags 114 and 116, respectively. In the other biological replication, the WT and Δ*bcactA* strains were labeled with iTRAQ tags 115 and 114, respectively. The labeled samples of each biological replication were separately pooled, vacuum dried, reconstituted in 0.1% formic acid, and submitted for nanoLC-MS/MS analysis.

The mass spectrometry (MS) analysis was performed using a nanoLC system (NanoLC-2D Ultra Plus; Eksigent, USA) coupled with a Triple TOF 5600 Plus mass spectrometer (AB Sciex) (48). The iTRAQ-labeled peptide samples were desalted with a 100-μm by 20-mm trap column and separated on an analytical 75-μm by 150-mm column packed with a Magic C_18_-AQ 5-μm, 200-Å phase (Michrom). The mobile phase A was 0.1% formic acid in water, while the mobile phase B was 0.1% formic acid in acetonitrile. Peptides were eluted in a linear gradient of 5% to 30% mobile phase B at a flow rate of 300 nl min^−1^. Precursor ions were selected across the mass range of 350 to 1,500 *m/z*^−1^ in high-resolution mode (>30,000). A maximum of 25 precursors per cycle from each MS spectrum were selected for fragmentation with 100-ms minimum accumulation time for each precursor. Tandem mass spectra were recorded in high-sensitivity mode (resolution, >15,000).

Protein identification and quantification were performed using the ProteinPilot 4.5 software (AB Sciex). Mass tolerance for fragment ions was automatically set by the software when using the Triple TOF 5600 Plus mass spectrometer. A database search was performed against the *B. cinerea* protein database with the following parameters: (i) sample type: iTRAQ 4-plex (peptide labeled); (ii) cysteine alkylation: MMTS; (iii) digestion: trypsin; (iv) instrument: Triple TOF 5600; (v) species: none; (vi) quantitate: yes; (vii) bias correction: yes; (viii) background correction: yes; (ix) search effort: thorough; (x) FDR analysis: yes. For iTRAQ quantitation, the peptide for quantification was automatically selected by the Pro Group algorithm (AB Sciex) to calculate the reporter peak area. A reverse database search strategy was applied to estimate the global FDR for peptide identification. Only proteins identified below the 1% global FDR were ultimately exported, and a 2-fold cutoff was used as a determinant for proteins showing a significant change in abundance. A Venn diagram was generated by a web-based Venn diagram software program (http://bioinfogp.cnb.csic.es/tools/venny/index.html), and a heat map (Pearson algorithm) was constructed using the PermutMatrix software (version 1.9.3).

### Phenotype analysis.

For radial growth assays, hyphal disks of the WT and mutants with a diameter of 1 mm were removed from the growing edges. The disks were excised using a cut pipette tip (200 μl, yellow; Axygen, CA, USA) with an inner diameter of 1 mm. Cultures were grown at 22°C, and measurements were taken daily from 1 to 3 days after inoculation. For sporulation assays, spores were harvested 10 days later using distilled water, and the suspension was filtered through four layers of gauze to remove mycelial fragments. The spores were counted with a hemocytometer under a microscope.

### Virulence assays.

Spores were harvested by adding sterile water to CM cultures and filtering the mixture through four layers of gauze to remove mycelial fragments. The density of the spore suspension was determined using a hemocytometer under a microscope. Infection assays in tomato and apple fruits were performed according to the methods described in our previous study (49). Tomato and apple fruits were sterilized by immersion in 2% sodium hypochlorite solution for 2 min, rinsed with tap water, and wounded to the same depth (approximately 4 mm) with a sterile nail. Conidia were adjusted to 1 × 10^4^ spores ml^−1^, and 10 μl was inoculated into the wounds of apples and tomatoes. Distilled water was used as negative control. Lesion formation was examined at 3 to 5 days after inoculation. For leaf infection assays, conidia were diluted to 2 × 10^5^ spores ml^−1^ with PDB, and 10 μl was dropped onto tomato leaves harvested from 4-week-old tomato plants. The leaves were placed in moist chambers at 22°C for lesion formation. All experiments were repeated three times, and 15 apples, nine tomatoes and six leaves were used in each replicate.

### Western blotting.

Total cytoplasmic proteins were extracted from 2-day-old mycelia cultured in PDB, according to methods described previously (29). Protein samples (20 μg) were separated by 12% SDS-PAGE and then electrotransferred onto a polyvinylidene difluoride (PVDF) membrane by semidry transfer. The membrane was blocked with 5% nonfat milk in phosphate-buffered saline with Tween 20 (PBST) buffer for 1.5 h at room temperature. Immunoblotting was conducted by incubation with a commercially available antibody of actin (Abmart; M20011), produced by immunizing mice with the human actin full-length protein. The immunoreactive bands were visualized by using a chemiluminescence detection kit (SuperSignal; Pierce Biotechnology). Histone 3 (H3) was used as a loading control.

### Statistical analysis of data.

Statistical differences in phenotype observation and virulence determination experiments were determined using the SPSS software (SPSS Inc., Chicago, IL, USA). One-way analysis of variance (ANOVA) and Tukey’s test were used, and a *P* value of <0.05 was considered statistically significant.

## References

[1] Bunnell T, Burbach B, Shimizu Y, Ervasti J 2011 β-Actin specifically controls cell growth, migration, and the G-actin pool. Mol Biol Cell 22:4047–4058. doi:10.1091/mbc.E11-06-0582. 21900491 PMC3204067

[2] Kandasamy MK, McKinney EC, Meagher RB 2002 Functional nonequivalency of actin isovariants in *Arabidopsis*. Mol Biol Cell 13:251–261. doi:10.1091/mbc.01-07-0342. 11809837 PMC65086

[3] Gilliland LU, Kandasamy MK, Pawloski LC, Meagher RB 2002 Both vegetative and reproductive actin isovariants complement the stunted root hair phenotype of the arabidopsis *act*2-1 mutation. Plant Physiol 130:2199–2209. doi:10.1104/pp.014068. 12481103 PMC166731

[4] Araujo-Bazán L, Peñalva MA, Espeso EA 2008 Preferential localization of the endocytic internalization machinery to hyphal tips underlies polarization of the actin cytoskeleton in *Aspergillus nidulans*. Mol Microbiol 67:891–905. doi:10.1111/j.1365-2958.2007.06102.x. 18179595

[5] Peñalva MÁ 2010 Endocytosis in filamentous fungi: Cinderella gets her reward. Curr Opin Microbiol 13:684–692. doi:10.1016/j.mib.2010.09.005. 20920884

[6] Shaw BD, Chung D-W, Wang C-L, Quintanilla LA, Upadhyay S 2011 A role for endocytic recycling in hyphal growth. Fungal Biol 115:541–546. doi:10.1016/j.funbio.2011.02.010. 21640317

[7] Takeshita N, Manck R, Grün N, de Vega SH, Fischer R 2014 Interdependence of the actin and the microtubule cytoskeleton during fungal growth. Curr Opin Microbiol 20:34–41. doi:10.1016/j.mib.2014.04.005. 24879477

[8] Fehrenbacher KL, Yang H-C, Gay AC, Huckaba TM, Pon LA 2004 Live cell imaging of mitochondrial movement along actin cables in budding yeast. Curr Biol 14:1996–2004. doi:10.1016/j.cub.2004.11.004. 15556861

[9] Taheri-Talesh N, Xiong Y, Oakley BR 2012 The functions of myosin II and myosin V homologs in tip growth and septation in *Aspergillus nidulans*. PLoS One 7:e31218. doi:10.1371/journal.pone.0031218. 22359575 PMC3281053

[10] Taheri-Talesh N, Horio T, Araujo-Bazán L, Dou X, Espeso EA, Peñalva MA, Osmani SA, Oakley BR 2008 The tip growth apparatus of *Aspergillus nidulans*. Mol Biol Cell 19:1439–1449. doi:10.1091/mbc.e07-05-0464. 18216285 PMC2291424

[11] Ryder LS, Dagdas YF, Mentlak TA, Kershaw MJ, Thornton CR, Schuster M, Chen J, Wang Z, Talbot NJ 2013 NADPH oxidases regulate septin-mediated cytoskeletal remodeling during plant infection by the rice blast fungus. Proc Natl Acad Sci U S A 110:3179–3184. doi:10.1073/pnas.1217470110. 23382235 PMC3581893

[12] Harris SD, Morrell JL, Hamer JE 1994 Identification and characterization of *Aspergillus nidulans* mutants defective in cytokinesis. Genetics 136:517–532. 8150280 10.1093/genetics/136.2.517 PMC1205805

[13] Rasmussen CG, Glass NL 2005 A Rho-type GTPase, rho-4, is required for septation in *Neurospora crassa*. Eukaryot Cell 4:1913–1925. doi:10.1128/EC.4.11.1913-1925.2005. 16278458 PMC1287859

[14] González-Rodríguez VE, Garrido C, Cantoral JM, Schumacher J 2016 The F-actin capping protein is required for hyphal growth and full virulence but is dispensable for septum formation in *Botrytis cinerea*. Fungal Biol 120:1225–1235. doi:10.1016/j.funbio.2016.07.007. 27647239

[15] Torralba S, Raudaskoski M, Pedregosa AM, Laborda F 1998 Effect of cytochalasin A on apical growth, actin cytoskeleton organization and enzyme secretion in *Aspergillus nidulans*. Microbiology 144:45–53. doi:10.1099/00221287-144-1-45. 9537763

[16] Dean R, van Kan JAL, Pretorius ZA, Hammond-Kosack KE, Pietro AD, Spanu PD, Rudd JJ, Dickman M, Kahmann R, Ellis J 2012 The top 10 fungal pathogens in molecular plant pathology. IMA Fungus 13:414–430. doi:10.1111/j.1364-3703.2011.00783.x. PMC6638784 22471698

[17] Weiberg A, Wang M, Lin FM, Zhao H, Zhang Z, Kaloshian I, Huang HD, Jin HL 2013 Fungal small RNAs suppress plant immunity by hijacking host RNA interference pathways. Science 342:118–123. doi:10.1126/science.1239705. 24092744 PMC4096153

[18] Fillinger S, Elad Y 2016 Botrytis—the fungus, the pathogen and its management in agricultural systems. Springer International Publishing, Cham, Switzerland.

[19] Girard V, Dieryckx C, Job C, Job D 2013 Secretomes: the fungal strike force. Proteomics 13:597–608. doi:10.1002/pmic.201200282. 23349114

[20] Qin GZ, Zong YY, Chen QL, Hua DL, Tian SP 2010 Inhibitory effect of boron against *Botrytis cinerea* on table grapes and its possible mechanisms of action. Int J Food Microbiol 138:145–150. doi:10.1016/j.ijfoodmicro.2009.12.018. 20060611

[21] Li BQ, Wang WH, Zong YY, Qin GZ, Tian SP 2012 Exploring pathogenic mechanisms of *Botrytis cinerea* secretome under different ambient pH based on comparative proteomic analysis. J Proteome Res 11:4249–4260. doi:10.1021/pr300365f. 22746291

[22] Zhang ZQ, Qin GZ, Li BQ, Tian SP 2014 Knocking out Bcsas1 in *Botrytis cinerea* impacts growth, development, and secretion of extracellular proteins, which decreases virulence. Mol Plant Microbe Interact 27:590–600. doi:10.1094/MPMI-10-13-0314-R. 24520899

[23] Schumacher J 2012 Tools for *Botrytis cinerea*: new expression vectors make the gray mold fungus more accessible to cell biology approaches. Fungal Genet Biol 49:483–497. doi:10.1016/j.fgb.2012.03.005. 22503771

[24] Qin GZ, Tian SP, Chan ZL, Li BQ 2007 Crucial role of antioxidant proteins and hydrolytic enzymes in pathogenicity of *Penicillium expansum* analysis based on proteomics approach. Mol Cell Proteomics 6:425–438. doi:10.1074/mcp.M600179-MCP200. 17194899

[25] Pradet-Balade B, Boulmé F, Beug H, Müllner EW, Garcia-Sanz JA 2001 Translation control: bridging the gap between genomics and proteomics? Trends Biochem Sci 26:225–229. doi:10.1016/S0968-0004(00)01776-X. 11295554

[26] Wang YY, Wang WH, Cai JH, Zhang YR, Qin GZ, Tian SP 2014 Tomato nuclear proteome reveals the involvement of specific E2 ubiquitin-conjugating enzymes in fruit ripening. Genome Biol 15:548. doi:10.1186/s13059-014-0548-2. 25464976 PMC4269173

[27] Liu RL, Wang YY, Qin GZ, Tian SP 2016 iTRAQ-based quantitative proteomic analysis reveals the role of the tonoplast in fruit senescence. J Proteomics 146:80–89. doi:10.1016/j.jprot.2016.06.031. 27371350

[28] van Kan JA, van’t Klooster JW, Wagemakers CA, Dees DC, van der Vlugt-Bergmans CJ 1997 Cutinase A of *Botrytis cinerea* is expressed, but not essential, during penetration of gerbera and tomato. Mol Plant Microbe Interact 10:30–38. doi:10.1094/MPMI.1997.10.1.30. 9002270

[29] Pollard TD, Cooper JA 2009 Actin, a central player in cell shape and movement. Science 326:1208–1212. doi:10.1126/science.1175862. 19965462 PMC3677050

[30] Rinnerthaler M, Büttner S, Laun P, Heeren G, Felder TK, Klinger H, Weinberger M, Stolze K, Grousl T, Hasek J, Benada O, Frydlova I, Klocker A, Simon-Nobbe B, Jansko B, Breitenbach-Koller H, Eisenberg T, Gourlay CW, Madeo F, Burhans WC, Breitenbach M 2012 Yno1p/Aim14p, a NADPH-oxidase ortholog, controls extramitochondrial reactive oxygen species generation, apoptosis, and actin cable formation in yeast. Proc Natl Acad Sci U S A 109:8658–8663. doi:10.1073/pnas.1201629109. 22586098 PMC3365156

[31] Li H, Zhang ZQ, He C, Qin GZ, Tian SP 2016 Comparative proteomics reveals the potential targets of BcNoxR, a putative regulatory subunit of NADPH oxidase of *Botrytis cinerea*. Mol Plant Microbe Interact 29:990–1003. doi:10.1094/MPMI-11-16-0227-R. 27898285

[32] Dominguez R, Holmes KC 2011 Actin structure and function. Annu Rev Biophys 40:169–186. doi:10.1146/annurev-biophys-042910-155359. 21314430 PMC3130349

[33] De Lisle RC 2015 Role of the actin cytoskeleton in acinar cell protein secretion. Pancreapedia: exocrine pancreas knowledge base. https://www.pancreapedia.org/reviews/role-of-actin-cytoskeleton-in-acinar-cell-protein-secretion.

[34] Choquer M, Fournier E, Kunz C, Levis C, Pradier J-M, Simon A, Viaud M 2007 *Botrytis cinerea* virulence factors: new insights into a necrotrophic and polyphageous pathogen. FEMS Microbiol Lett 277:1–10. doi:10.1111/j.1574-6968.2007.00930.x. 17986079

[35] ten Have A, Dekkers E, Kay J, Phylip LH, van Kan JA 2004 An aspartic proteinase gene family in the filamentous fungus *Botrytis cinerea* contains members with novel features. Microbiology 150:2475–2489. doi:10.1099/mic.0.27058-0. 15256589

[36] Kubicek CP, Starr TL, Glass NL 2014 Plant cell wall–degrading enzymes and their secretion in plant-pathogenic fungi. Annu Rev Phytopathol 52:427–451. doi:10.1146/annurev-phyto-102313-045831. 25001456

[37] Chai Y, Zhao M 2017 iTRAQ-based quantitative proteomic analysis of the inhibitory effects of polysaccharides from viscum coloratum (Kom.) nakai on HepG2 cells. Sci Rep 7:4596. doi:10.1038/s41598-017-04417-x. 28676664 PMC5496916

[38] van Kan JA 2006 Licensed to kill: the lifestyle of a necrotrophic plant pathogen. Trends Plant Sci 11:247–253. doi:10.1016/j.tplants.2006.03.005. 16616579

[39] Rolke Y, Liu S, Quidde T, Williamson B, Schouten A, Weltring KM, Siewers V, Tenberge KB, Tudzynski B, Tudzynski P 2004 Functional analysis of H_2_O_2_-generating systems in *Botrytis cinerea*: the major Cu-Zn-superoxide dismutase (BCSOD1) contributes to virulence on French bean, whereas a glucose oxidase (BCGOD1) is dispensable. Mol Plant Pathol 5:17–27. doi:10.1111/j.1364-3703.2004.00201.x. 20565578

[40] Tian SP, Torres R, Ballester A, Li BQ, Vilanova L, González-Candelas L 2016 Molecular aspects in pathogen-fruit interactions: virulence and resistance. Postharvest Biol Technol 122:11–21. doi:10.1016/j.postharvbio.2016.04.018.

[41] Shah P, Atwood JA, Orlando R, El Mubarek H, Podila GK, Davis MR 2009 Comparative proteomic analysis of *Botrytis cinerea* secretome. J Proteome Res 8:1123–1130. doi:10.1021/pr8003002. 19140674

[42] Espino JJ, Gutiérrez-Sánchez G, Brito N, Shah P, Orlando R, González C 2010 The *Botrytis cinerea* early secretome. Proteomics 10:3020–3034. doi:10.1002/pmic.201000037. 20564262 PMC3983782

[43] Have A, Mulder W, Visser J, van Kan JA 1998 The endopolygalacturonase gene Bcpg1 is required for full virulence of *Botrytis cinerea*. Mol Plant Microbe Interact 11:1009–1016. doi:10.1094/MPMI.1998.11.10.1009. 9768518

[44] Kars I, Krooshof GH, Wagemakers L, Joosten R, Benen JA, Van Kan JA 2005 Necrotizing activity of five *Botrytis cinerea* endopolygalacturonases produced in *Pichia pastoris*. Plant J 43:213–225. doi:10.1111/j.1365-313X.2005.02436.x. 15998308

[45] Brito N, Espino JJ, González C 2006 The endo-β-1,4-xylanase Xyn11A is required for virulence in *Botrytis cinerea*. Mol Plant Microbe Interact 19:25–32. doi:10.1094/MPMI-19-0025. 16404950

[46] Zhang LS, Thiewes H, van Kan JA 2011 The -galacturonic acid catabolic pathway in Botrytis cinerea. Fungal Genet Biol 48:990–997. doi:10.1016/j.fgb.2011.06.002. 21683149

[47] Bradford MM 1976 A rapid and sensitive method for the quantitation of microgram quantities of protein utilizing the principle of protein-dye binding. Anal Biochem 72:248–254. doi:10.1016/0003-2697(76)90527-3. 942051

[48] Wang WH, Cai JH, Wang PW, Tian SP, Qin GZ 2017 Post-transcriptional regulation of fruit ripening and disease resistance in tomato by the vacuolar protease SlVPE3. Genome Biol 18:47. doi:10.1186/s13059-017-1178-2. 28270225 PMC5341188

[49] Zhang ZQ, Qin GZ, Li BQ, Tian SP 2014 Infection assays of tomato and apple fruit by the fungal pathogen *Botrytis cinerea*. Bio Protoc 4:e1311. doi:10.21769/BioProtoc.1311.

