# Supplementary figures and images for: Actin Is Required for Cellular Development and Virulence of Botrytis cinerea via the Mediation of Secretory Proteins
Source: mSystems. 2020 Feb 25;5(1):e00732-19. doi: 10.1128/mSystems.00732-19 (PMC7043344; doi:10.1128/mSystems.00732-19)

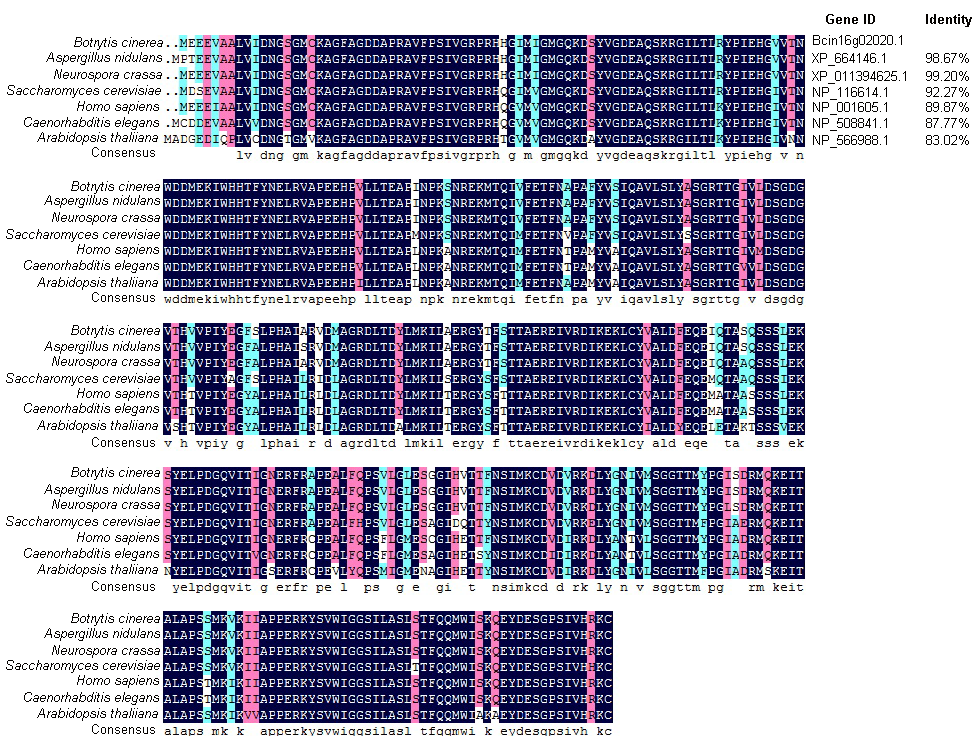

Supplement: FIG S1 [file mSystems.00732-19-sf001.tif]

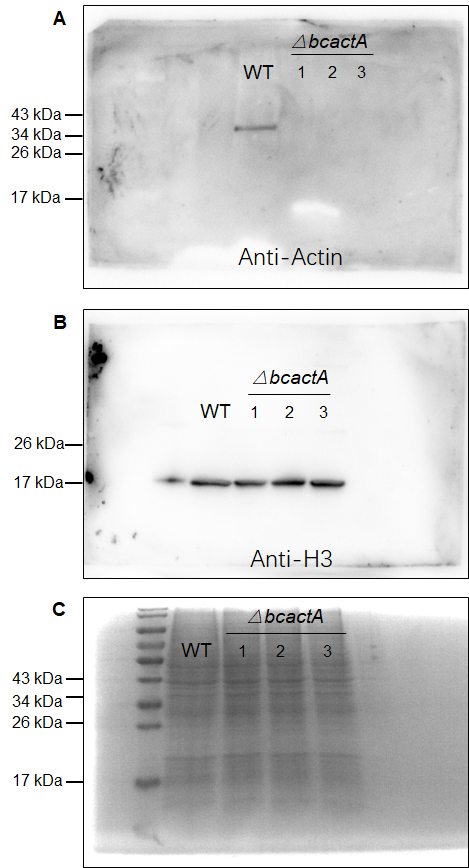

Supplement: FIG S2 [file mSystems.00732-19-sf002.tif]

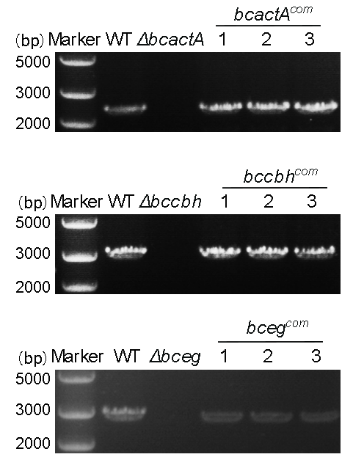

Supplement: FIG S3 [file mSystems.00732-19-sf003.tif]

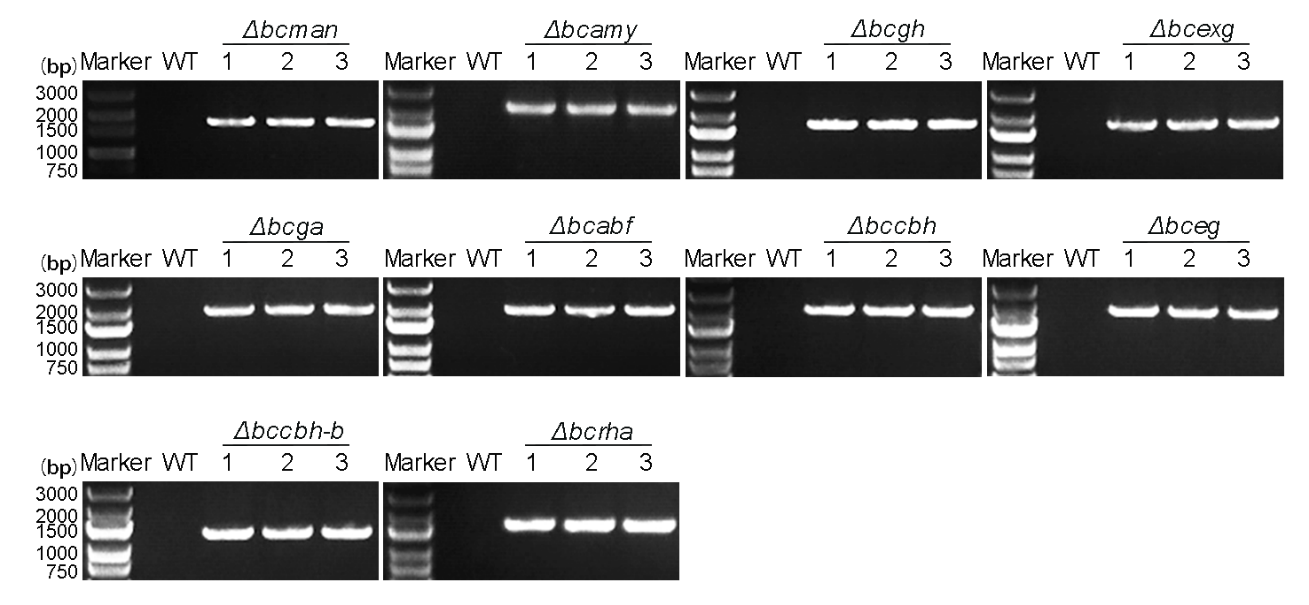

Supplement: FIG S4 [file mSystems.00732-19-sf004.tif]

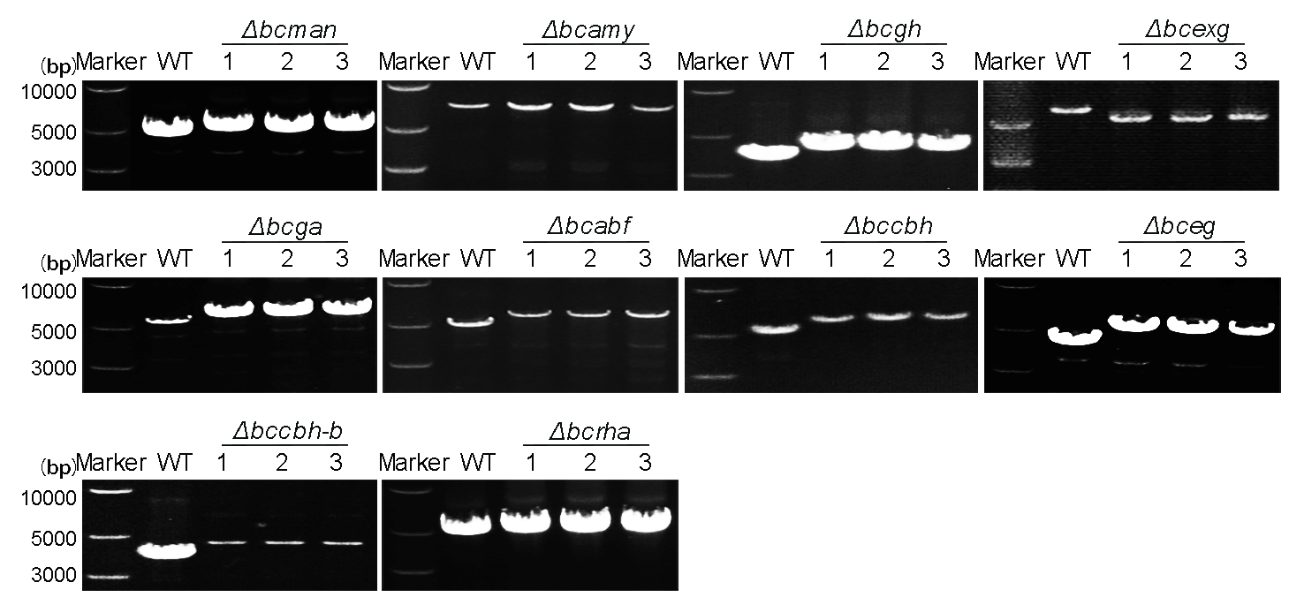

Supplement: FIG S5 [file mSystems.00732-19-sf005.tif]

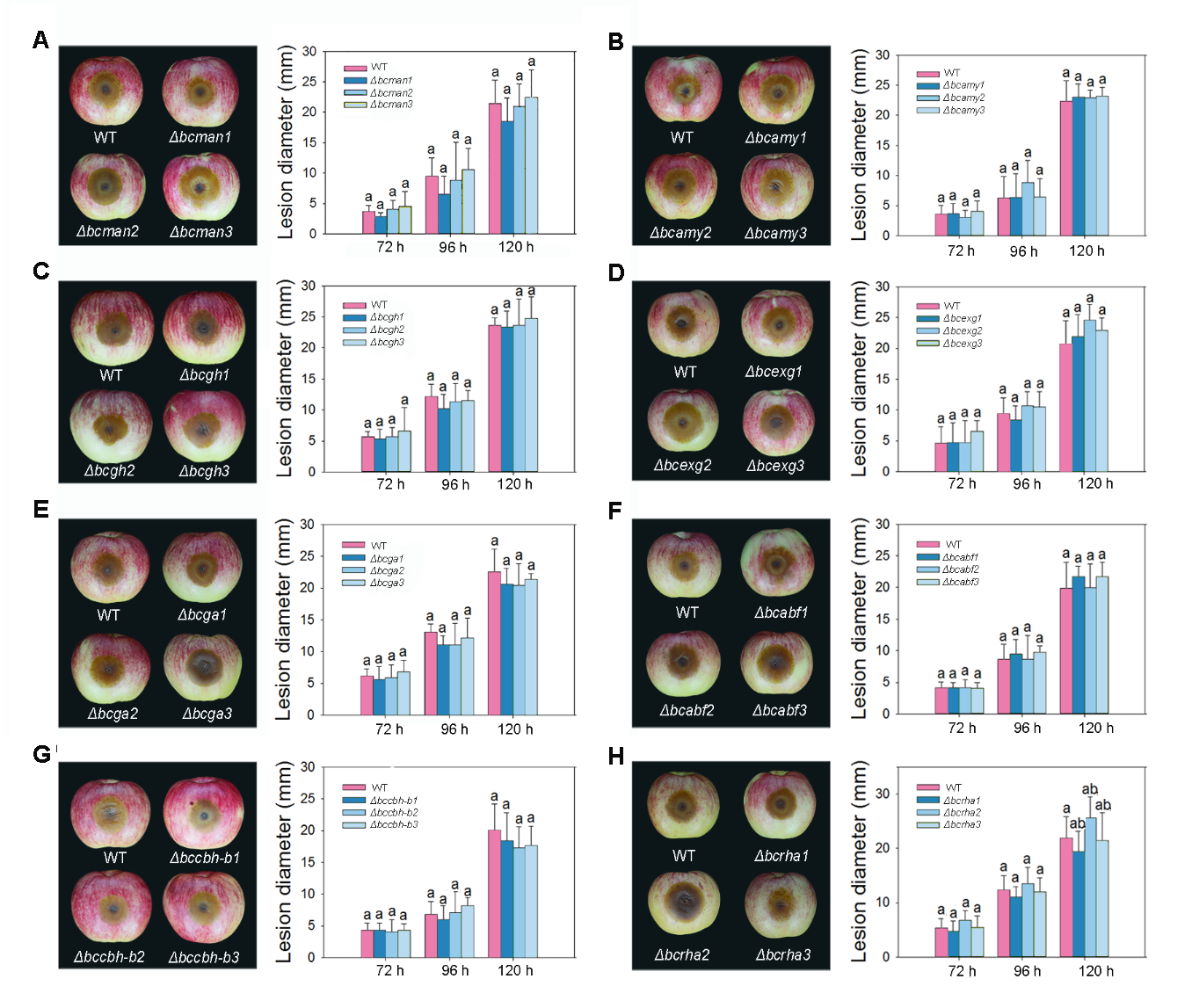

Supplement: FIG S6 [file mSystems.00732-19-sf006.tif]

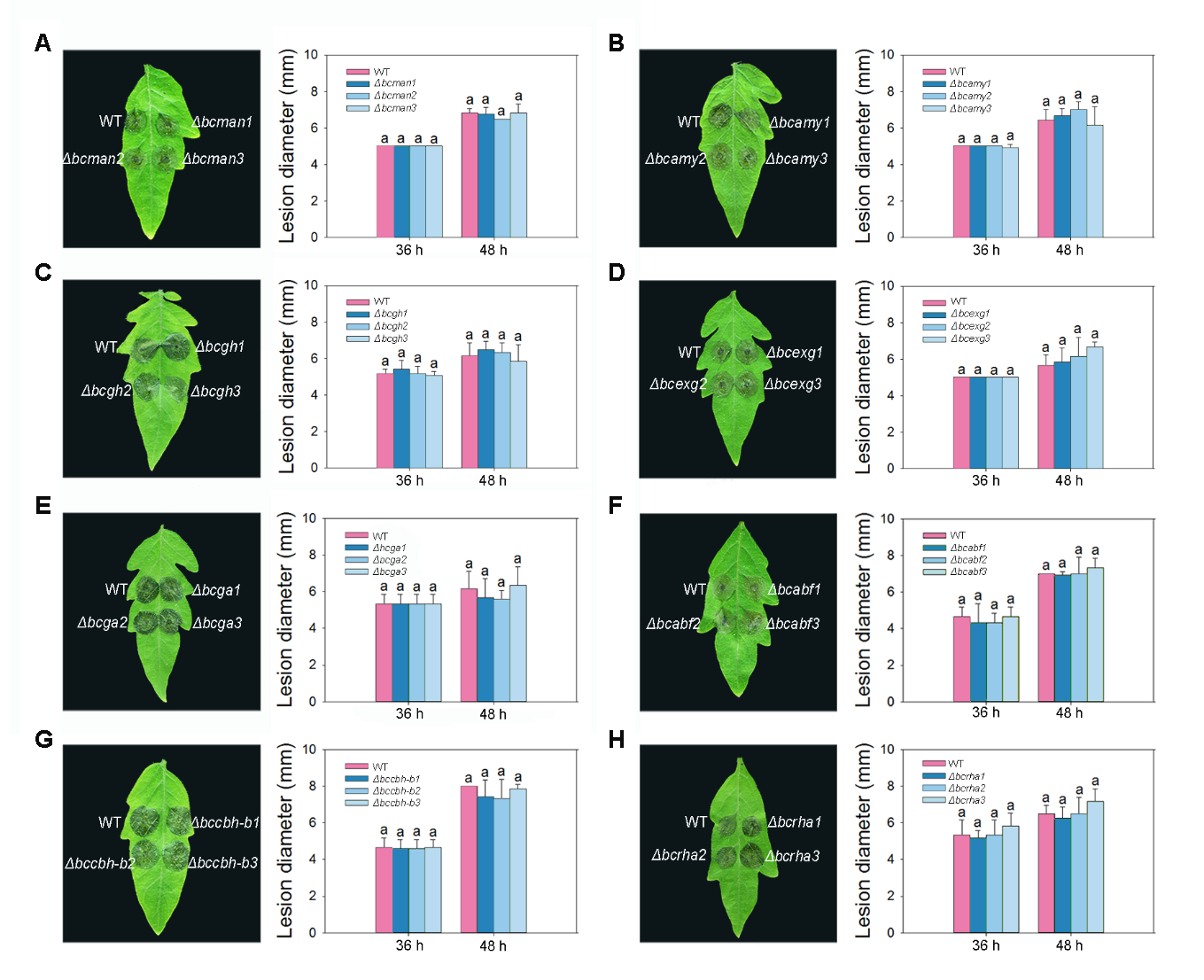

Supplement: FIG S7 [file mSystems.00732-19-sf007.tif]

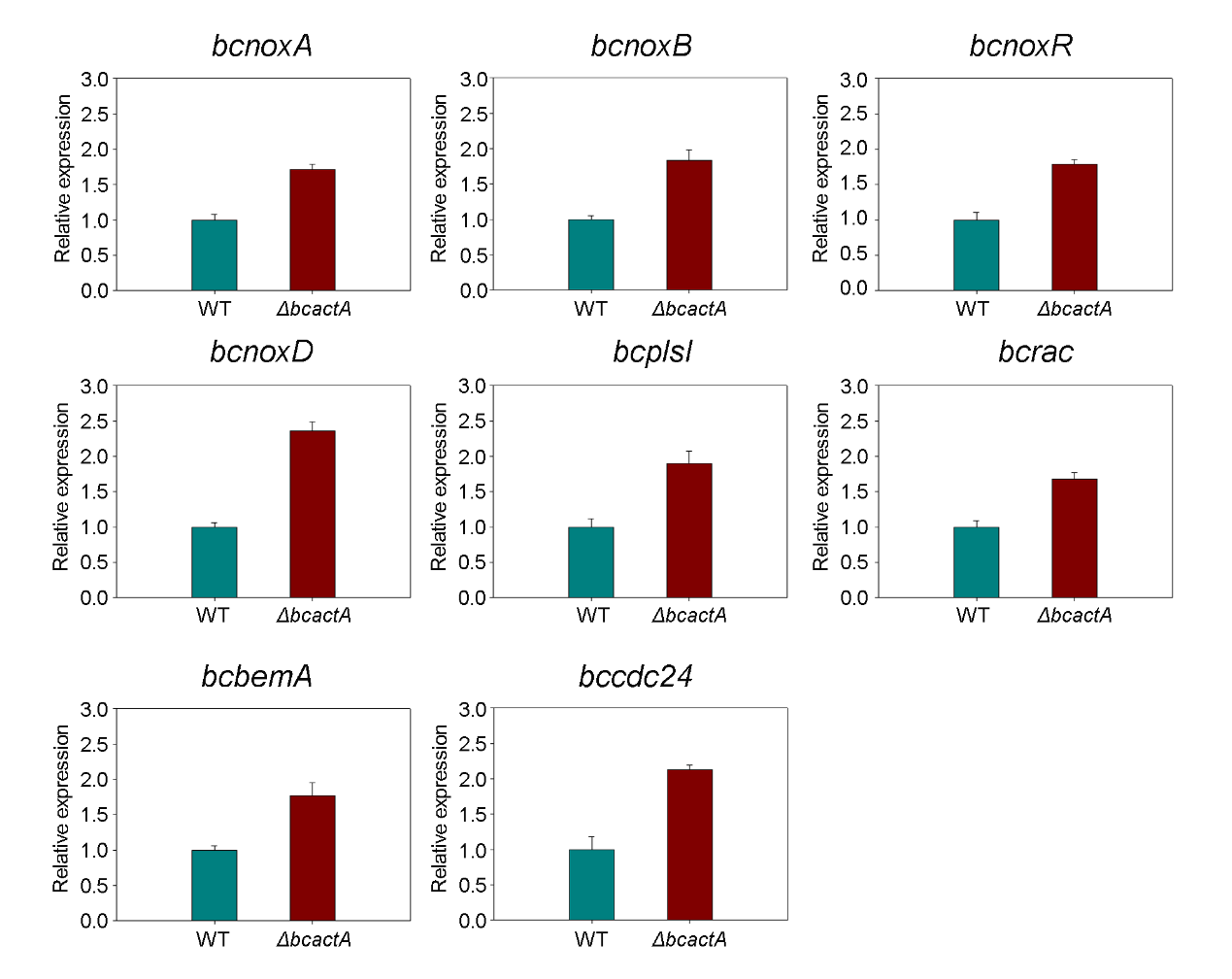

Supplement: FIG S8 [file mSystems.00732-19-sf008.tif]
