# Supplementary material for: Actin Is Required for Cellular Development and Virulence of Botrytis cinerea via the Mediation of Secretory Proteins
Source: mSystems. 2020 Feb 25;5(1):e00732-19. doi: 10.1128/mSystems.00732-19 (PMC7043344; doi:10.1128/mSystems.00732-19)
Supplement: TABLE S1 [file mSystems.00732-19-st001.docx]

**Table S1.** Primers used in vector construction.

| **Primer** | **Sequence (5’-3’)** | **Application** | **Enzyme,** **annealing temperature and thermal cycle** | **Expected length of the amplicons (bp)** |
| --- | --- | --- | --- | --- |
| *bcactA*-L-up | AAAGGCGCCCTGGCTGGAAATCTAACGC | Amplification of 5' flank | KAPA HiFi  65 °C | 1035 |
| *bcactA*-L-down | AGGCGCGCCGGTTGATAAATTAAGACG |  | 35 cycle |  |
| *bcactA*-R-up | ACGCGTCGACGGTATGTAATTATCAATGG | Amplification of 3' flank | KAPA HiFi  65 °C | 913 |
| *bcactA*-R-down | CGGGATCCAGACAGGACCATCATAGCAG |  | 35 cycle |  |
| *bcactA*-homo-up | CGGAGTACATAGAGCAGAG | Diagnostic PCR for homozygotes, amplify with *bcactA*-R-down | KAPA HiFi  65 °C  35 cycle | 3725 for wild type  4464 for the mutant |
| HPH-det | CGGGATCCCGACCGGGATCTTAGTGAT | Diagnostic PCR, amplify with *gene*-homo-up | Taq PCR mix  49 °C  35 cycle | 1674, 1700, 1949, 1735, 1742, 1928, 1927, 1940, 1989, 1613 and 1713 for *bcactA* and 10 CWDE genes, respectively |
| *bcactA*-com-up-SpeI | TGCTCCTTCAATATCACTAGTTGGGAACGATAGACAGAG | Complementary vector construction | KAPA HiFi  65 °C | 4310 |
| *bcactA*-com-down-EcoRI | TTTCTTTGAATTATAGAATTCCCCAAGCCTAAACTCGTAC |  | 35 cycle |  |
| *bccbh*-com-up-SpeI | TGCTCCTTCAATATCACTAGT CACGAGAACAAAGAGGACCCG | Complementary vector construction | KAPA HiFi  65 °C  35 cycle | 4127 |
| *bccbh*-com-down-EcoRI | TTTCTTTGAATTATAGAATTC GATTCCGAGACCTTGTTTCC |  |  |  |
| *bceg*-com-up-SpeI | TGCTCCTTCAATATCACTAGTGTGAATCGGTGCATCTAAGGA | Complementary vector construction | KAPA HiFi  65 °C  35 cycle | 3488 |
| *bceg*-com-down-NotI | ATCTACATACGCTAAGCGGCCGCTCGAACAAAGGGGTAAAAGGT |  |  |  |
| *bcman*-homo-up | TGTCTGGGGCTTGCTGAAGATAC | Diagnostic PCR for homozygotes | KAPA HiFi  65 °C | 4548 for wild type  4997 for the mutant |
| *bcman*-homo-down | TTGCTACGTGCGGAAGAATTAGG |  | 35 cycle |  |
| *bcman*-L-up-NarI | AAAGGCGCCATGGTCCGTGACCTTCTCC | Amplification of 5' flank | KAPA HiFi  65 °C  35 cycle | 1119 |
| *bcman*-L-down-AscI | AAAGGCGCGCCTGTAATATTAAACCCAAGA |  |  |  |
| *bcman*-R-up-BamHI | AAAGGATCCTCAACTAGTTGAAATTTTG | Amplification of 3' flank | KAPA HiFi  65 °C  35 cycle | 924 |
| *bcman*-R-down-KpnI | AAAGGTACCTCTCAACCCTGTCCCTAA |  |  |  |
| *bcamy*-homo-up | AGGTGTTTCCTCGGCTTTCAGTG | Diagnostic PCR for homozygotes | KAPA HiFi  65 °C  35 cycle | 5775 for wild type  5708 for the mutant |
| *bcamy*-homo-down | GTAGAGTTGTGGAAGCAATCGAGC |  |  |  |
| *bcamy*-L-up-NarI | AAAGGCGCCTATCATGTGCGTTCACGG | Amplification of 5' flank | KAPA HiFi  65 °C | 835 |
| *bcamy*-L-down-AscI | AAAGGCGCGCCTTTGAGATGAGAGAGGGG |  | 35 cycle |  |
| *bcamy*-R-up-BamHI | AAAGGATCCAGGCGGAAAGGGAAAGGAG | Amplification of 3' flank | KAPA HiFi  65 °C | 926 |
| *bcamy*-R-down-KpnI | AAAGGTACCCAGCCGAGATTGGGAGTA |  | 35 cycle |  |
| *bcgh*-homo-up | GAATCAGGGACAAGGAAGT | Diagnostic PCR for homozygotes | KAPA HiFi  65 °C | 4049 for wild type  5198 for the mutant |
| *bcgh*-homo-down | CTATGGGATTGTGGAGGGTGAGT |  | 35 cycle |  |
| *bcgh*-L-up-HindIII | ACCAAGCTTTGTGGAGGCTATGGACTTG | Amplification of 5' flank | KAPA HiFi  65 °C | 1021 |
| *bcgh*-L-down-AscI | AAAGGCGCGCC AGCTGAATGTTTTGAAATATCG |  | 35 cycle |  |
| *bcgh*-R-up-BamHI | AAAGGATCCTATTTCTTCGGATTTTTG | Amplification of 3' flank | KAPA HiFi  65 °C | 1135 |
| *bcgh*-R-down-KpnI | AAAGGTACCTCGGCGTCCTCATTTACG |  | 35 cycle |  |
| *bcexg*-homo-up | CCTTCGTATTGGGTGGTG | Diagnostic PCR for homozygotes | KAPA HiFi  65 °C | 5690 for wild type  5187 for the mutant |
| *bcexg*-homo-down | CCTGATCTGGATGGAACTTACGG |  | 35 cycle |  |
| *bcexg*-L-up-NarI | AAAGGCGCCCCTCGGTCTAAATGGAAG | Amplification of 5' flank | KAPA HiFi  65 °C | 1023 |
| *bcexg*-L-down-AscI | AAAGGCGCGCCTTTGGACCCCATTAGTAAC |  | 35 cycle |  |
| *bcexg*-R-up-BamHI | AAAGGATCC GGTATCTGGCATTTTCAATG | Amplification of 3' flank | KAPA HiFi  65 °C | 889 |
| *bcexg*-R-down-KpnI | AAAGGTACCGGATAAATCCAGCCCAGAG |  | 35 cycle |  |
| *bcga*-homo-up | ATGGTTCGCTCACGCACTG | Diagnostic PCR for homozygotes | KAPA HiFi  65 °C | 5523 for wild type  5780 for the mutant |
| *bcga*-homo-down | TCCATCCTTGCCCACGAGCCTAC |  | 35 cycle |  |
| *bcga*-L-up-NarI | AAAGGCGCCTTGTCTATGCCGCCGTTGTC | Amplification of 5' flank | KAPA HiFi  65 °C | 1147 |
| *bcga*-L-down-AscI | AAAGGCGCGCC GGCTATTTATATTGGCGAG |  | 35 cycle |  |
| *bcga*-R-up-BamHI | AAAGGATCC TCGTGAGGATTTTTCTCG | Amplification of 3' flank | KAPA HiFi  65 °C | 822 |
| *bcga*-R-down-KpnI | AAAGGTACCAGGGCATCTCCTTAACAGTCG |  | 35 cycle |  |
| *bcabf*-homo-up | TGCTGCGTTGTCATTTCC | Diagnostic PCR for homozygotes | KAPA HiFi  65 °C | 5005 for wild type  5864 for the mutant |
| *bcabf*-homo-down | CCCTCCTCAACTCTTCCACCCTC |  | 35 cycle |  |
| *bcabf*-L-up- AscI | AAAGGCGCGCCCCGCAACATTTTAGGGAC | Amplification of 5' flank | KAPA HiFi  65 °C | 1100 |
| *bcabf*-L-down-AscI | AAAGGCGCGCCATCTAATCACTTTTGTGGCG |  | 35 cycle |  |
| *bcabf*-R-up-BamHI | AAAGGATCCATTCTTATCCGAATACTCCC | Amplification of 3' flank | KAPA HiFi  65 °C | 947 |
| *bcabf*-R-down-KpnI | AAAGGTACCCTGCTATCAGAACGGAGC |  | 35 cycle |  |
| *bccbh*-homo-up | TATGTGGTCTGAGAATCGGGGTTG | Diagnostic PCR for homozygotes | KAPA HiFi  65 °C | 5132 for wild type  5985 for the mutant |
| *bccbh*-homo-down | GATGGGCTACTTCTGCGTGTTAC |  | 35 cycle |  |
| *bccbh*-L-up-NarI | AAAGGCGCCCGGATGGTTCTATTTAGCC | Amplification of 5' flank | KAPA HiFi  65 °C  35 cycle | 947 |
| *bccbh*-L-down-AscI | AAAGGCGCGCCTTTGAAAGGATGTTGGTG |  |  |  |
| *bccbh*-R-up-BamHI | AAAGGATCCATTGTTTCCCTCGGGAAC | Amplification of 3' flank | KAPA HiFi  65 °C  35 cycle | 985 |
| *bccbh*-R-down-KpnI | AAAGGTACCAGCACTCAGCCAACTCAT |  |  |  |
| *bceg*-homo-up | TGAGTCAACGGGGTAATG | Diagnostic PCR for homozygotes | KAPA HiFi  65 °C | 4269 for wild type  5318 for the mutant |
| *bceg*-homo-down | TATCGACGTAGACAGCGGGGTTC |  | 35 cycle |  |
| *bceg*-L-up-NarI | AAAGGCGCCGTGTCGGGACGGATAAAC | Amplification of 5' flank | KAPA HiFi  65 °C | 941 |
| *bceg*-L-down-AscI | AAAGGCGCGCCGATGAATTAGTATTGTTTG |  | 35 cycle |  |
| *bceg*-R-up-BamHI | AAAGGATCCATGAAATGAGTTGTGATTATGG | Amplification of 3' flank | KAPA HiFi  65 °C | 1030 |
| *bceg*-R-down-KpnI | AAAGGTACCTCAGACTAACGACTAAACCC |  | 35 cycle |  |
| *bccbh-b* -homo-up | AACTCTTGGGGTATCGTC | Diagnostic PCR for homozygotes | KAPA HiFi  65 °C | 3871 for wild type  4891 for the mutant |
| *bccbh-b*- homo-down | GACTTTCTCCCTTCGTTCGTCTG |  | 35 cycle |  |
| *bccbh-b*-L-up-NarI | AAAGGCGCCTTTTGGTCCTGTGACTGC | Amplification of 5' flank | KAPA HiFi  65 °C | 1013 |
| *bccbh-b*-L-down- NarI | AAAGGCGCCGATGAGTTTCTGTCAGATTC |  | 35 cycle |  |
| *bccbh-b*-R-up-BamHI | AAAGGATCCGGGATATATGGATTCAAG | Amplification of 3' flank | KAPA HiFi  65 °C | 883 |
| *bccbh-b*-R-down-KpnI | AAAGGTACCCATGGGTCACTTGTAAGG |  | 35 cycle |  |
| *bcrha*-homo-up | GACTACTCCTCGGGCTGAACATA | Diagnostic PCR for homozygotes | KAPA HiFi  65 °C | 4796 for wild type  5100 for the mutant |
| *bcrha*-homo-down | CATGCTCCATCCTTGTAGGTTAC |  | 35 cycle |  |
| *bcrha*-L-up-HindIII | ACCAAGCTTTGTAACTTTGCGGAGACC | Amplification of 5' flank | KAPA HiFi  65 °C | 909 |
| *bcrha*-L-down-AscI | AAAGGCGCGCCATTTTTGCCAGCTTGGAAC |  | 35 cycle |  |
| *bcrha*-R-up-BamHI | AAAGGATCCCGACTGGCCTGATTTTATTTCC | Amplification of 3' flank | KAPA HiFi  65 °C | 847 |
| *bcrha*-R-down-KpnI | AAAGGTACCCAGCAAAACTTGGGGACA |  | 35 cycle |  |
